# PD-1 blockade improves Kupffer cell bacterial clearance in acute liver injury

**DOI:** 10.1172/JCI140196

**Published:** 2021-02-15

**Authors:** Evangelos Triantafyllou, Cathrin L.C. Gudd, Marie-Anne Mawhin, Hannah C. Husbyn, Francesca M. Trovato, Matthew K. Siggins, Thomas O’Connor, Hiromi Kudo, Sujit K. Mukherjee, Julia A. Wendon, Christine Bernsmeier, Robert D. Goldin, Marina Botto, Wafa Khamri, Mark J.W. McPhail, Lucia A. Possamai, Kevin J. Woollard, Charalambos G. Antoniades, Mark R. Thursz

**Affiliations:** 1 Department of Metabolism, Digestion and Reproduction, Section of Hepatology and Gastroenterology, and; 2 Department of Immunology and Inflammation, Centre for Inflammatory Disease, Imperial College London, London, United Kingdom.; 3 Division of Transplantation Immunology and Mucosal Biology, King’s College London, London, United Kingdom.; 4 Department of Infectious Disease, and; 5 Department of Metabolism, Digestion and Reproduction, Section of Pathology, Imperial College London, London, United Kingdom.; 6 Department of Biomedicine, University of Basel and University Centre for Gastrointestinal and Liver Diseases, Basel, Switzerland.

**Keywords:** Hepatology, Immunology, Innate immunity, Macrophages

## Abstract

Patients with acute liver failure (ALF) have systemic innate immune suppression and increased susceptibility to infections. Programmed cell death 1 (PD-1) expression by macrophages has been associated with immune suppression during sepsis and cancer. We therefore examined the role of the programmed cell death 1/programmed death ligand 1 (PD-1/PD-L1) pathway in regulating Kupffer cell (KC) inflammatory and antimicrobial responses in acetaminophen-induced (APAP-induced) acute liver injury. Using intravital imaging and flow cytometry, we found impaired KC bacterial clearance and systemic bacterial dissemination in mice with liver injury. We detected increased PD-1 and PD-L1 expression in KCs and lymphocyte subsets, respectively, during injury resolution. Gene expression profiling of PD-1^+^ KCs revealed an immune-suppressive profile and reduced pathogen responses. Compared with WT mice, PD-1–deficient mice and anti–PD-1–treated mice with liver injury showed improved KC bacterial clearance, a reduced tissue bacterial load, and protection from sepsis. Blood samples from patients with ALF revealed enhanced PD-1 and PD-L1 expression by monocytes and lymphocytes, respectively, and that soluble PD-L1 plasma levels could predict outcomes and sepsis. PD-1 in vitro blockade restored monocyte functionality. Our study describes a role for the PD-1/PD-L1 axis in suppressing KC and monocyte antimicrobial responses after liver injury and identifies anti–PD-1 immunotherapy as a strategy to reduce infection susceptibility in ALF.

## Graphical abstract

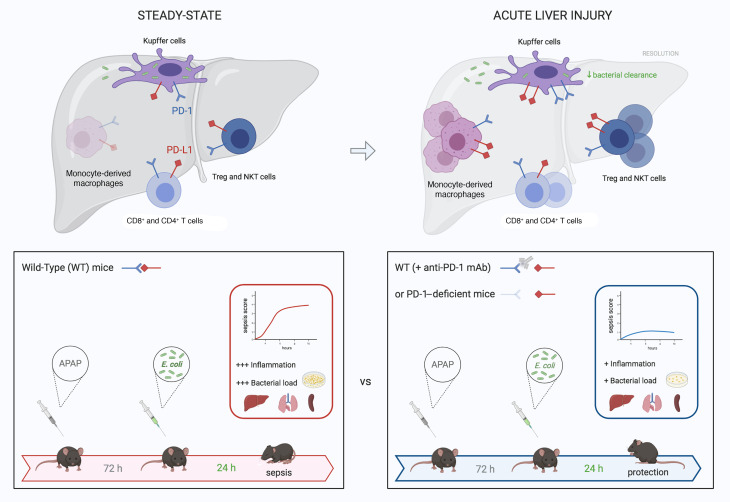

## Introduction

Acute liver failure (ALF) is a rare clinical syndrome in which coagulopathy, jaundice, and hepatic encephalopathy arise in the context of acute hepatic injury without preexisting chronic liver disease (CLD) (1, 2). Acetaminophen (APAP) overdose is the most common cause of ALF in the United Kingdom and the United States (1, 2). Immune dysfunction is central to the pathogenesis of ALF. Although the initiating event in ALF is overwhelming hepatocyte death, mortality occurs as a result of profound activation of hepatic and systemic inflammatory responses and the associated complications of multiorgan failure, immune paresis, and sepsis (3).

Bacterial infections are common complications of ALF, occurring late (>5 days after hospital admission) in 35%–40% of patients and are considered a leading cause of mortality (3–5). This is a consequence of ALF-related immune paresis and the invasive nature of critical care support that predisposes patients to nosocomial infections (3). Our group has previously demonstrated defects in both innate and adaptive systemic immunity in ALF (4–8). Monocytes of patients with ALF are hyporesponsive to microbial challenge and show reduced proinflammatory cytokine secretion, impaired *Escherichia* *coli* bacterial uptake, and an M2-like phenotype that favors the resolution of inflammation (4–6, 8).

Macrophages are central to maintaining liver homeostasis but are equally relevant in responses to injury or infection (3, 9). Liver-resident macrophages, or Kupffer cells (KCs), have an embryonic origin and a capacity for self-renewal (3, 9). KCs reside within liver sinusoids and function as the dominant intravascular phagocytes with a fundamental role in the clearance of blood-borne bacteria, a process that is critical for the prevention of disseminated infections (10–13). After APAP-induced liver injury in mice, a reduction in KCs occurs 24–48 hours after APAP, and full recovery takes place by self-renewal (6, 14–16). The total macrophage pool is expanded during resolution (APAP, 72 h) via recruitment of bone marrow monocytes that differentiate in situ into monocyte-derived macrophages (MoMFs) (14–17). Human and mouse studies indicate that KCs and MoMFs have both tissue-destructive and pro-restorative/tissue repair functions in APAP injury (6, 14–16). However, the role of KCs in mediating antimicrobial responses after liver injury remains unexplored.

Programmed cell death 1 (PD-1) plays an essential role in balancing protective immunity and immunopathology, homeostasis, and tolerance. However, during chronic infections and cancer, it can limit protective immunity (18). PD-1 is a major regulator of T cell responses, expressed on activated T cells, B cells, and Tregs (18, 19). PD-1 interacts with its ligands, PD-L1 and PDL-2, and their engagement triggers downstream signaling. Immune checkpoint inhibition using mAbs against PD-1/PD-L1 has revolutionized cancer treatment, with significant clinical success in some patients (18, 19). In an effort to increase the therapeutic reach of targeting this pathway, the role of PD-1 has been examined in DCs, NK cells, and innate lymphoid cells (20).

Expression in myeloid cells of PD-1 and its potential for therapeutic targeting have also started to be appreciated. PD-1 can be induced in monocytes and macrophages through TLR signaling (21, 22). Furthermore, PD-1 expression by macrophages plays a pathologic role by suppressing innate inflammatory responses in sepsis and inhibiting phagocytosis in active tuberculosis (23, 24). In the context of cancer, PD-1 expression in macrophages inversely correlates with M1-like polarization and the phagocytic potency of tumor-associated macrophages (TAMs) against tumor (25, 26). PD-1 expression in myeloid cells was very recently shown to dampen antitumor immunity by regulating myeloid cell lineage fate commitment and function (27). However, the role of this pathway in regulating macrophage responses during acute liver injury remains unknown.

In this study, we first aimed to examine KC bacterial clearance in mice with APAP-induced acute liver injury. Second, we sought to investigate the role of the PD-1/PD-L1 pathway in regulating KC inflammatory and antimicrobial responses after injury. This was further assessed in blood and liver tissue samples from patients with ALF. We provide evidence that following injury, KCs display impaired bacterial clearance, a suppressive effect that is mediated via the PD-1/PD-L1 axis. Our work identifies PD-1–targeted immunotherapy as a strategy to reduce susceptibility to infections in liver failure.

## Results

### Reduced KC bacterial clearance and increased bacterial dissemination in mice with acute liver injury.

**Figure 1:**
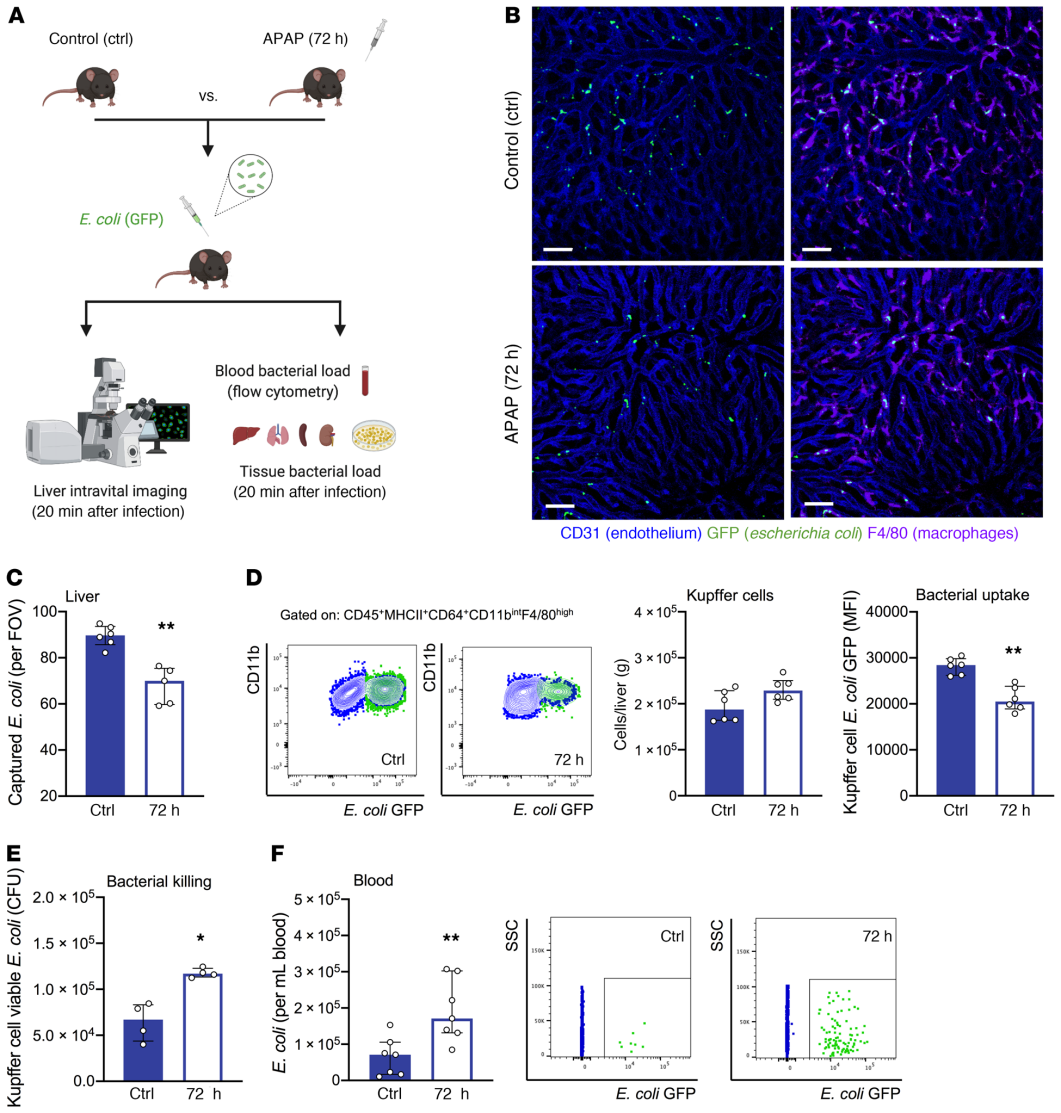
KC bacterial clearance is reduced in mice with acute liver injury. Baseline (control [ctrl]) and APAP-treated (72 h) mice were intravenously challenged with *E*. *coli*. (**A**) Schematic of experimental *E*. *coli* challenge in mice. (**B**) Representative intravital liver images (20 min after infection). Macrophages and endothelial cells were stained with fluorescently labeled anti-F4/80 (purple) and anti-CD31 (blue) antibodies, respectively; GFP^+^ *E*. *coli* (green). Scale bars: 50 μm. (**C**) Intravital imaging analysis shows the amount of macrophage-captured *E*. *coli* per FOV (*n* = 5–6 per group). (**D**) Representative flow plots of KC (blue) *E*. *coli* (green) capture measured by flow cytometry. Dot plots show KC numbers and *E*. *coli* uptake as measured by flow cytometry (*n* = 6 per group). (**E**) Sorted KCs from control and APAP-treated (72 h) mice were in vitro challenged with *E*. *coli* (1:100 ratio, 60 min). In vitro bacterial killing of KC-phagocytosed *E*. *coli* was evaluated by measuring viable *E*. *coli* CFU recovered from KC lysates (*n* = 4 per group). (**F**) Representative dot plots and number of free GFP^+^ *E*. *coli* in blood measured by flow cytometry (*n* = 7 per group). SSC, side scatter. Results are from 3 (**B**–**D**, and **F**) and 2 (**E**) independent experiments. Each symbol represents an individual mouse. Data are presented as the median with the IQR. *P < 0.05 and **P < 0.01, by Mann-Whitney *U* test.

To examine the functional status of KCs in mediating bacterial clearance in the liver, we studied untreated (control) and APAP-treated (72 h: resolution phase) mice following systemic *E*. *coli* infection (Figure 1A). Using liver intravital imaging to visualize the dynamic interactions between KCs and circulating *E*. *coli* bacteria, we found that *E*. *coli* were arrested in the liver within 20 minutes of infection (Figure 1, B and C), in line with the crucial role of KCs in clearing blood-borne infections (10–13). *E*. *coli* bacteria colocalized with F4/80-labeled KCs (Figure 1B), while 3D reconstitution with increased KC transparency confirmed that captured *E*. *coli* were inside KCs (Supplemental Videos 1 and 2; supplemental material available online with this article; https://doi.org/10.1172/JCI140196DS1).

Compared with controls, APAP-treated mice showed fewer captured *E*. *coli* in the liver (20 min after infection, Figure 1, B and C). KC numbers were similar between study groups (Figure 1D and Supplemental Figure 1A), suggesting that the decreased *E*. *coli* capture after liver injury was due to a qualitative defect in KC phagocytosis rather than a quantitative deficiency in KCs. To test this, we performed flow cytometry and found reduced *E*. *coli* uptake in KCs isolated from infected APAP-treated mice compared with controls (Figure 1D). We further examined bacteria internalization by KCs using intravital imaging in mice challenged with pHrodo *E*. *coli*, a pH-sensitive assay indicating phagolysosome acidification, which confirmed a reduced KC bacterial uptake capacity in APAP-treated mice (Supplemental Figure 1, B and C). In addition, KCs from APAP-treated mice were characterized by a decrease in bacterial killing ability, as indicated by the greater number of viable *E*. *coli* recovered from KCs (Figure 1E and Supplemental Figure 1E).

We further quantified the number of bacteria in the blood and different organs (20 min after infection). Compared with controls, APAP-treated mice displayed significantly higher blood *E*. *coli* counts (Figure 1F) and increased bacterial burden in the spleen, lungs, and kidneys (Supplemental Figure 1, D and E). Together, these data suggest reduced KC bacterial clearance and systemic bacterial dissemination in mice with acute liver injury.

### Increased PD-1 and PD-L1 expression in KCs after acute liver injury in mice.

**Figure 2:**
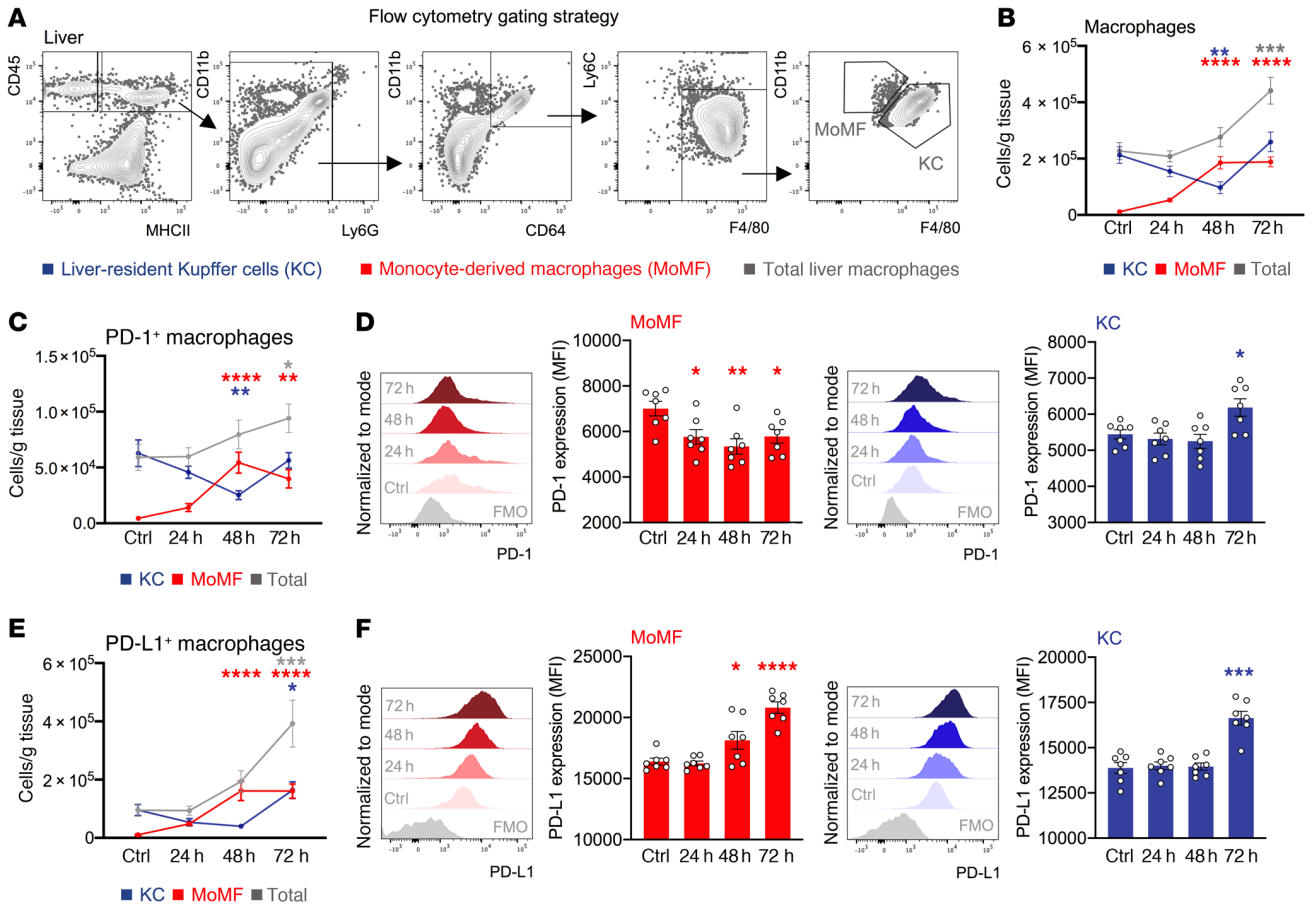
PD-1 and PD-L1 expression in KCs is increased during the resolution of acute liver injury. Hepatic nonparenchymal cells were isolated from livers of baseline (control) and APAP-treated (24 h, 48 h, or 72 h) WT mice. Phenotypic characterization of liver CD45^+^ leukocytes was done by flow cytometry. (**A**) Representative flow cytometric gating strategy used to identify MoMFs and liver-resident KCs. (**B**) Number of MoMFs (red), KCs (blue), and total macrophages (gray) (*n* = 10–12 per group). (**C**) Number of PD-1^+^ MoMFs (red), PD-1^+^ KCs (blue), and PD-1^+^ total liver macrophages (gray) (*n* *=* 7 per group). (**D**) Representative histograms and data showing PD-1 expression (MFI) by MoMFs and KCs (*n* *=* 7 per group). (**E**) Number of PD-L1^+^ MoMFs (red), PD-L1^+^ KCs (blue), and PD-L1^+^ total liver macrophages (gray) (*n* *=* 7 per group). (**F**) Representative histograms and data showing PD-L1 expression (MFI) of MoMFs and KCs (*n* = 7 per group). Results are from 3 (**B**) and 2 (**C**–**F**) independent experiments. Each symbol represents an individual mouse. Data are presented as the mean ± SEM. *P < 0.05, **P < 0.01, ***P < 0.001, and ****P < 0.0001, by 1-way ANOVA (compared with control). FMO, fluorescence minus one.

Given the implication of the PD-1/PD-L1 axis in macrophage suppression during sepsis and cancer (23, 26), we measured the levels of PD-1 and PD-L1 expression by macrophages during murine acute liver injury. We observed peak injury at 24 hours and resolution of inflammation within 72 hours of APAP treatment (refs. 6, 14 and Supplemental Figure 1F). Flow cytometric analysis of liver KCs and MoMFs (Figure 2A) showed a reduction of KCs at 48 hours with full recovery by 72 hours, as previously reported (14–16), whereas liver recruitment of MoMFs contributed to the expansion of total macrophages at 72 hours (Figure 2B and Supplemental Figure 2A). Both PD-1 and PD-L1 were expressed in KCs and MoMFs at baseline (Figure 2, C–F), and the total number of PD-1^+^ and PD-L1^+^ liver macrophages increased after injury (Figure 2, C and E, and Supplemental Figure 2, B and C). Notably, PD-1 and PD-L1 expression was increased in KCs during the resolution phase, whereas only PD-L1 expression was upregulated in MoMFs (Figure 2, D and F). In line with these data, liver *Pdl1* mRNA was upregulated at 72 hours (Supplemental Figure 1G).

### Increased PD-L1 expression of lymphocyte subsets after acute liver injury in mice.

**Figure 3:**
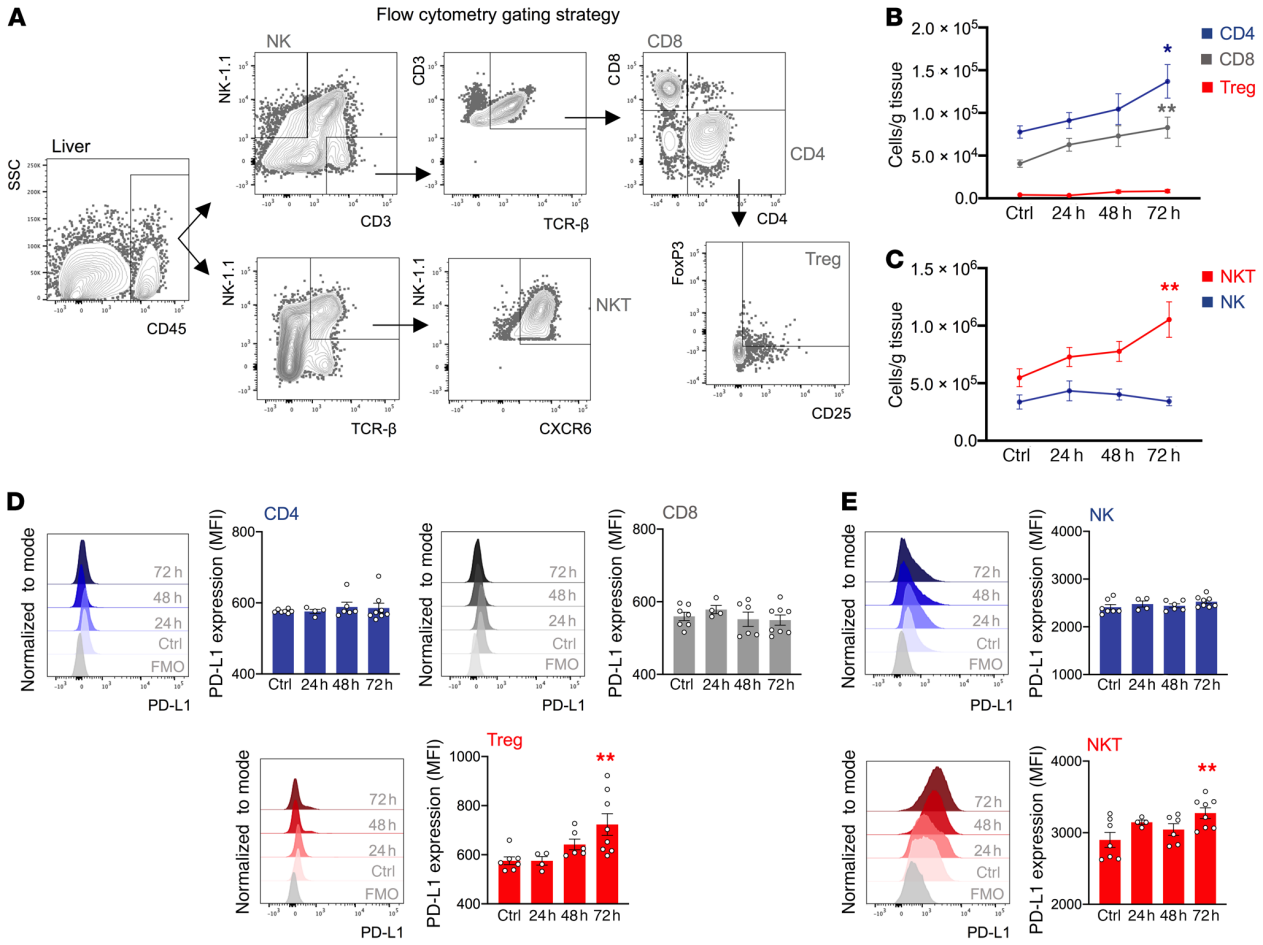
PD-L1 expression of lymphocyte subsets is increased during the resolution of acute liver injury. Hepatic nonparenchymal cells were isolated from livers of baseline (control) and APAP-treated (24 h, 48 h, or 72 h) WT mice. Phenotypic characterization of liver CD45^+^ leukocytes was done by flow cytometry. (**A**) Representative flow cytometric gating strategy used to identify CD4^+^ Τ cells, CD8^+^ Τ cells, Tregs, NK cells, and NKT cells. (**B**) Number of CD4^+^ T cells (blue), CD8^+^ T cells (gray), and Tregs (red) per gram of tissue (*n* = 8–12 per group). (**C**) Number of NK (blue) and NKT (red) cells per gram of tissue (*n* = 8–12 per group). (**D**) Representative histograms and data showing PD-L1 expression (MFI) of CD4^+^ T cells, CD8^+^ T cells, and Tregs (*n* = 4–8 per group). (**E**) Representative histograms and data showing PD-L1 expression (MFI) of NK and NKT cells (*n* = 4–8 per group). Results are from 3 (**B** and **C**) and 2 (**D** and **E**) independent experiments. Each symbol represents an individual mouse. Data are presented as the mean ± SEM. *P < 0.05 and **P < 0.01, by 1-way ANOVA (compared with control).

PD-1 signaling inhibits T cell activation, while PD-L1^+^ T cells can also induce tolerogenic macrophages via PD-1 ligation (28). To explore this in our context, we determined the PD-1 and PD-L1 expression levels in lymphocyte subsets during murine acute liver injury. Flow cytometric analysis revealed an expansion of liver CD4^+^ T cells, CD8^+^ T cells, and Tregs during the resolution phase (72 h) (Figure 3, A and B, and Supplemental Figure 2D). PD-1 expression in these cell subsets remained unaltered, whereas PD-L1 expression in Tregs was upregulated 72 hours after APAP (Figure 3D and Supplemental Figure 2F). In addition, flow cytometric analysis of NK and NKT cells revealed an expansion of NKTs during resolution (Figure 3, A and C, and Supplemental Figure 2E). NK cell PD-1 and PD-L1 expression remained unaltered (Figure 3E and Supplemental Figure 2G), whereas PD-L1 expression in NKT cells was upregulated at 72 hours (Figure 3E). In line with this, liver tissue mRNA levels of *Pdl1* and the lymphocyte recruitment chemokines *Cxcl10* and *Cxcl16* were upregulated at 72 hours (Supplemental Figure 1G).

### PD-1–expressing KCs exhibit an immune-suppressive profile in acute liver injury.

**Figure 4:**
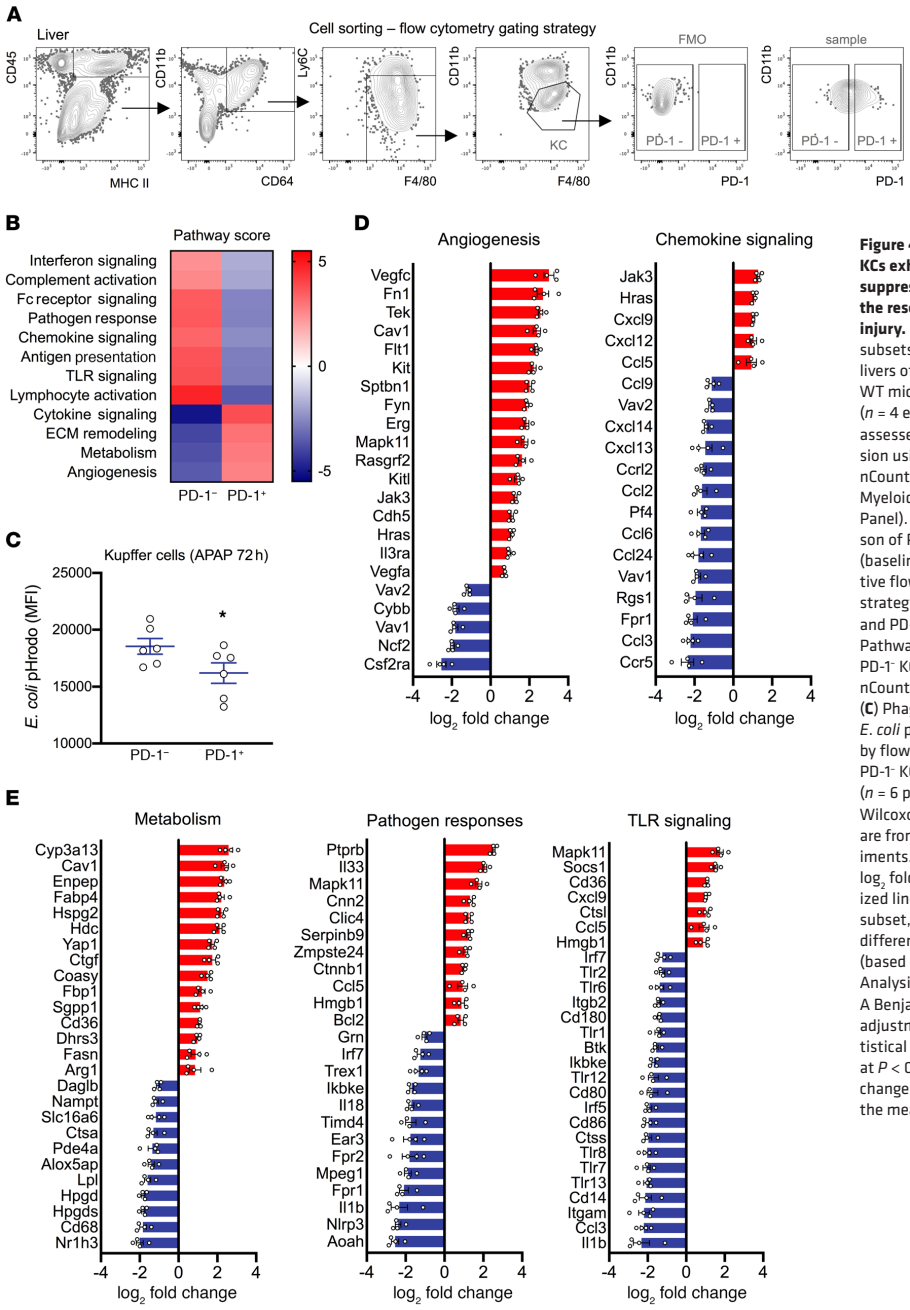
PD-1–expressing KCs exhibit an immune-suppressive profile during the resolution of acute liver injury. PD-1^+^ and PD-1^–^ KC subsets were sorted from livers of APAP-treated (72 h) WT mice using flow cytometry (*n* = 4 each). KC lysates were assessed for mRNA expression using the NanoString nCounter system (Mouse Myeloid Innate Immunity Panel). Data show comparison of PD-1^+^ with PD-1^–^ cells (baseline). (**A**) Representative flow cytometric gating strategy used to sort the PD-1^+^ and PD-1^–^ cell subsets. (**B**) Pathway scoring of PD-1^+^ and PD-1^–^ KCs performed using nCounter Advanced Analysis. (**C**) Phagocytosis (uptake) of *E*. *coli* pHrodo was assessed by flow cytometry in PD-1^+^ and PD-1^–^ KC subsets (APAP, 72 h) (*n* = 6 per group). *P < 0.05, by Wilcoxon paired test. Results are from 2 independent experiments. (**D** and **E**) Data show log_2_ fold change of normalized linear count data (PD-1^+^ subset, *n* = 4) for significantly differentially expressed genes (based on nCounter Advanced Analysis) in various pathways. A Benjamini-Hochberg *P* value adjustment was applied. Statistical significance was set at *P* < 0.05 and a 2-fold linear change. Data are presented as the mean ± SEM.

To fully characterize the PD-1^+^ KCs during resolution of the injury, flow cytometry–sorted PD-1^+^ and PD-1^–^ KCs (72 h after APAP) were subjected to mRNA expression analysis (Figure 4A, Supplemental Figure 3, and Supplemental Data File 1). Compared with PD-1^–^ KCs, PD-1^+^ KCs had increased scores for angiogenesis, metabolism, extracellular matrix (ECM) remodeling, and cytokine signaling (Figure 4B and Supplemental Figure 3B). In contrast, pathway scores for lymphocyte activation, antigen presentation, pathogen responses, and chemokine, IFN, TLR, and Fc receptor signaling were reduced in PD-1^+^ KCs (Figure 4B and Supplemental Figure 3B).

PD-1^+^ KCs exhibited upregulation of M2-like (*Arg1*, *Cd36*, *Cd206*, *Cdh1*, *Csf1*, *Fn1*) and downregulation of M1-like (*Irf5*, *Il1b*, *Il18*) macrophage polarization genes (Figure 4, D and E, and Supplemental Figure 3). They also had high expression of the suppressors of cytokine signaling *Socs1* and *Socs3*. Compared with PD-1^–^ KCs, PD-1^+^ KCs were characterized by the downregulation of genes related to lymphocyte regulation (*Btk*, *CD64*, *Cd80*, *Cd86*, *Syk*), complement activation, TLR signaling (*Itgam*, *Cd14*, *Cd80*, *Cd86*, *Btk*, *Irf5*, *Irf7*), and pathogen responses (*Aoah*, *Nlrp3*, *Il1b*, *Fpr1*, *Mpeg1*, *Ncf2*, *Cybb*) (Figure 4, D and E, and Supplemental Figure 3). To confirm the latter, flow cytometry–sorted PD-1^+^ and PD-1^–^ KCs were subjected to an *E*. *coli* phagocytosis assay. PD-1^+^ KCs showed reduced *E*. *coli* uptake, providing a functional correlate of the gene expression trends (Figure 4C). These data indicate that PD-1 expression by KCs is associated with an immune-suppressive profile and impaired antimicrobial functions in acute liver injury.

### PD-1^–/–^ mice with acute liver injury show efficient KC bacterial clearance and protection from sepsis.

**Figure 5:**
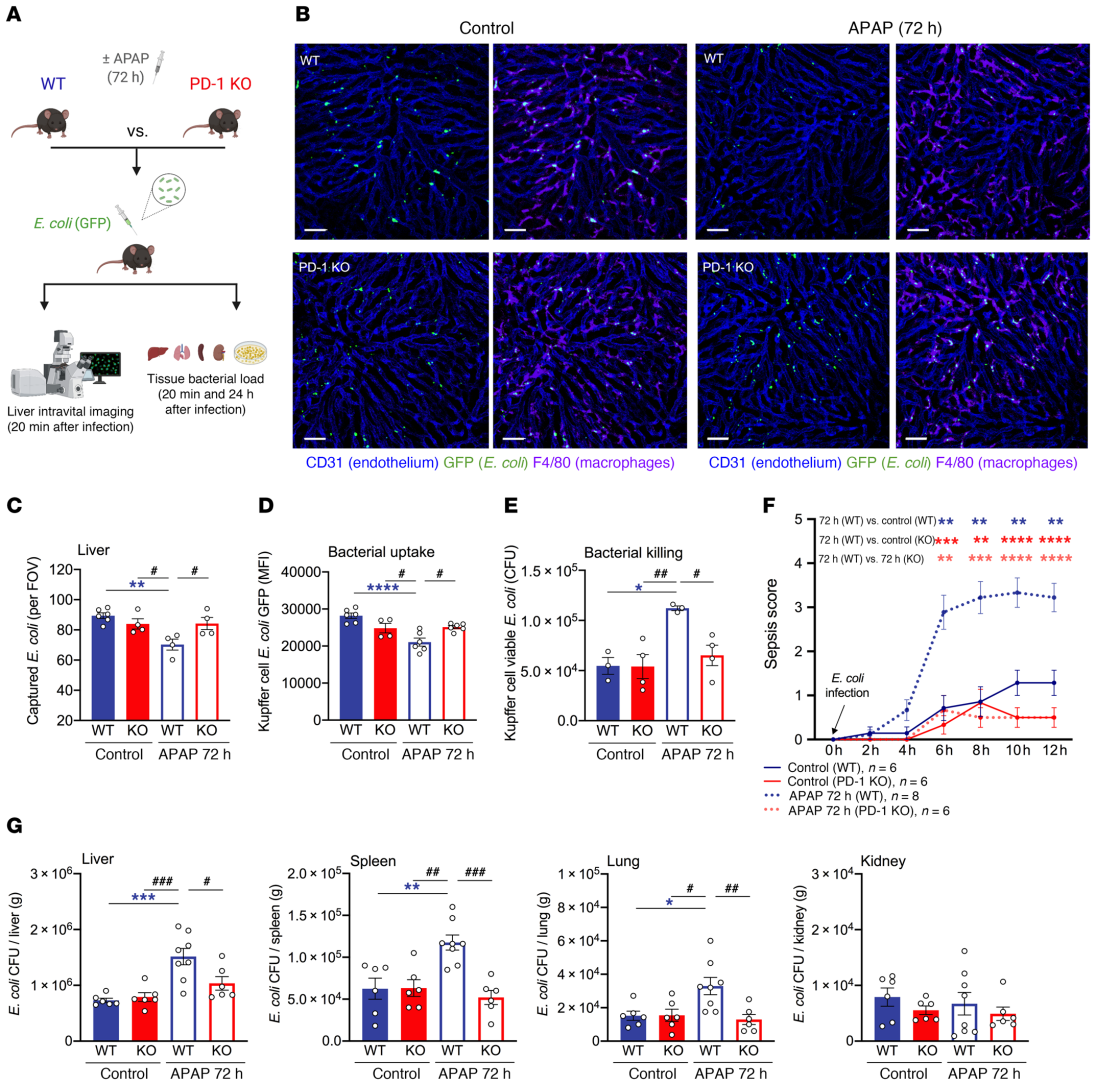
PD-1 deficiency improves KC bacterial clearance and confers protection from sepsis in mice with acute liver injury. Baseline (control) and APAP-treated (72 h) WT and PD-1–deficient (PD-1 KO) mice were intravenously challenged with *E*. *coli*. (**A**) Schematic of experimental *E*. *coli* challenge. (**B**) Representative intravital liver images (20 min after infection). Macrophages and endothelial cells were stained with fluorescently labeled anti-F4/80 (purple) and anti-CD31 (blue) antibodies, respectively; GFP^+^ *E*. *coli* (green). Scale bars: 50 μm. (**C**) Intravital liver imaging analysis showing the amount of macrophage-captured *E*. *coli* per FOV (*n* = 4–6 per group). (**D**) KC *E*. *coli* uptake measured by flow cytometry (*n* = 4–6 per group). (**E**) Sorted KCs from control or APAP-treated (72 h) WT and PD-1^–/–^ mice were in vitro challenged with *E*. *coli* (1:100 ratio, 60 min). In vitro bacterial killing of KC phagocytosed *E*. *coli* was evaluated by measuring viable *E*. *coli* CFU recovered from KC lysates (*n* = 3–4 per group). (**F**) Mouse sepsis scores over time following *E*. *coli* infection (*n* = 6–8 per group). (**G**) CFU analysis of bacterial burden in the liver, spleen, lungs, and kidneys 24 hours after infection (*n* = 6–8 per group). Results are from 3 (**B**–**D**, **F**, and **G**) and 2 (**E**) independent experiments. Each symbol represents an individual mouse. Data are presented as the mean ± SEM. * or ^#^*P* < 0.05, ** or ^##^*P* < 0.01, *** or ^###^*P* < 0.001, ****P < 0.0001, by 1-way ANOVA (**C**–**E** and **G**) or 2-way, repeated-measures ANOVA (**F**).

To determine whether the PD-1/PD-L1 axis may alter the inflammatory and antimicrobial responses of KCs after injury, we studied control and APAP-treated WT and PD-1^–/–^ mice (Supplemental Figure 4A). Compared with WT mice, PD-1^–/–^ mice had similar ALT levels but reduced hepatic necrosis scores 48 hours and 72 hours after APAP treatment (Supplemental Figure 4, B and C) and showed no differences in blood immune cells or liver monocytes and macrophages (Supplemental Figure 4, D–F, and Supplemental Figure 5, A and B). PD-1^–/–^ mice also had more CD8^+^ T cells in the liver, failed to show expanded PD-L1^hi^ NKT cells at resolution, and had reduced liver tissue *Pdl1,* *Cxcl10*, and *Cxcl16* mRNA levels (Supplemental Figure 5, D and E, and Supplemental Figure 4H). Intravital imaging and flow cytometric analysis revealed comparable KC bacterial capture in control WT and PD-1^–/–^ animals 20 minutes after *E. coli* infection (Figure 5, A–D). In contrast to APAP-treated WT mice, which had reduced KC *E*. *coli* capture and killing, APAP-treated PD-1^–/–^ mice showed a preserved KC phagocytic capacity (Figure 5, B–E) and a lower tissue bacterial burden, suggesting a lack of systemic bacterial dissemination (20 min after infection, Supplemental Figure 1E).

We further sought to evaluate disease severity at later time points after *E*. *coli* infection using an established experimental sepsis score (29) and by assessing the tissue bacterial burden and sepsis-related inflammatory markers 24 hours after infection (29–31). Compared with control WT mice, APAP-treated WT mice had significantly higher sepsis scores after infection (Figure 5F) and an increased bacterial load in the liver, spleen, and lungs (Figure 5G). In contrast to APAP-treated WT mice, control and APAP-treated PD-1^–/–^ mice had lower sepsis scores after infection (Figure 5F) and a lower bacterial load in the liver, spleen, and lungs (Figure 5G). In line with these data, APAP-treated WT mice showed an augmented inflammatory response following *E*. *coli* infection. Compared with APAP-treated PD-1^–/–^ mice, APAP-treated WT mice had a higher fold change of C-reactive protein (CRP) and lactate levels (Supplemental Figure 6, A and B) and a cytokine profile that favored a proinflammatory imbalance, as indicated by increased sepsis severity markers (29–31) such as IL-6 and a higher IL-6/IL-10 ratio (Supplemental Figure 6, C–F).

### PD-1 blockade improves KC bacterial clearance in mice with acute liver injury and confers protection from sepsis.

**Figure 6:**
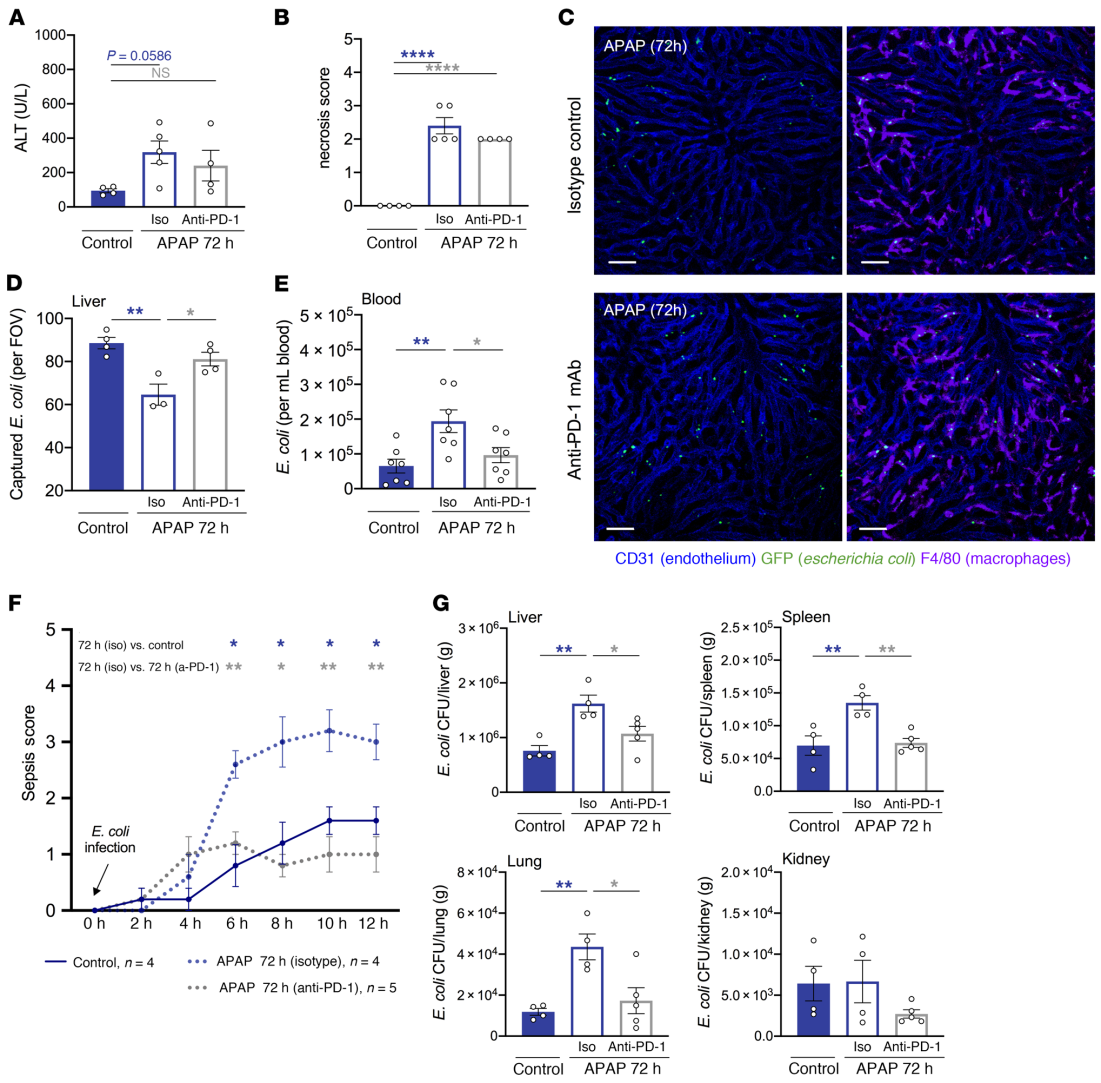
PD-1 blockade improves KC bacterial clearance and confers protection from sepsis in mice with acute liver injury. Baseline (control) and APAP-treated (72 h) WT mice, dosed with isotype control (iso) or anti–PD-1 mAb 48 hours after APAP treatment, were intravenously challenged with *E*. *coli*. (**A**) Plasma alanine transaminase (ALT) levels (U/L) and (**B**) hepatic necrosis scores (*n* = 4–5 per group). (**C**) Representative intravital liver images from APAP-treated (72 h) mice (20 min after infection). Macrophages and endothelial cells were stained with fluorescently labeled anti-F4/80 (purple) and anti-CD31 (blue) antibodies, respectively; GFP^+^ *E*. *coli* (green). Scale bars: 50 μm. (**D**) Intravital liver imaging analysis showing the amount of macrophage-captured *E*. *coli* per FOV (*n* = 3–4 per group). (**E**) Number of free *E*. *coli* in blood measured by flow cytometry (*n* = 7 per group). (**F**) Mouse sepsis scores over time following *E*. *coli* infection (*n* = 4–5 per group). (**G**) CFU analysis showing bacterial burden in the liver, spleen, lungs, and kidneys 24 hours after infection (*n* = 4–5 per group). Results in **A**–**G** are for 2 independent experiments. Each symbol represents an individual mouse. Data are presented as the mean ± SEM. *P < 0.05, **P < 0.01, and ****P < 0.0001, by 1-way ANOVA (**A**–**E** and **G**) or 2-way, repeated-measures ANOVA (**F**).

We next questioned whether in vivo blockade of the PD-1/PD-L1 pathway could improve KC bacterial clearance and prevent sepsis development in mice with liver injury. For this purpose, APAP-treated mice were dosed with a blocking anti–PD-1 Ab or an isotype control 48 hours after APAP treatment (Supplemental Figure 7A). Of note, liver injury indices and proportions of liver immune cell subset were similar between groups (Figure 6, A and B, and Supplemental Figure 7B). Compared with isotype-treated mice, anti–PD-1–treated mice showed enhanced KC bacterial capture in the liver (Figure 6, C and D, and Supplemental Figure 7, C and D), lower blood *E*. *coli* counts (Figure 6E), and reduced tissue bacterial burden (20 min after infection, Supplemental Figure 1E). Later time-point disease monitoring revealed that, compared with isotype-treated mice, anti–PD-1–treated mice had lower sepsis scores after infection (Figure 6F) and a reduced bacterial load in the liver, spleen, and lungs (24 h after infection, Figure 6G), as was seen in PD-1^–/–^ mice (Figure 5, F and G). Moreover, anti–PD-1–treated infected mice had unaltered CRP levels, lactate levels, and IL-6/IL-10 ratios compared with control mice (Supplemental Figure 6, C–F). Taken together, these findings demonstrate that PD-1 deficiency or anti–PD-1 therapy in mice with liver injury is associated with improved KC bacterial clearance, reduced tissue bacterial burden, and protection from sepsis after infection.

### Increased lymphocyte PD-L1 expression and soluble PD-L1 plasma levels in patients with ALF.

**Figure 7:**
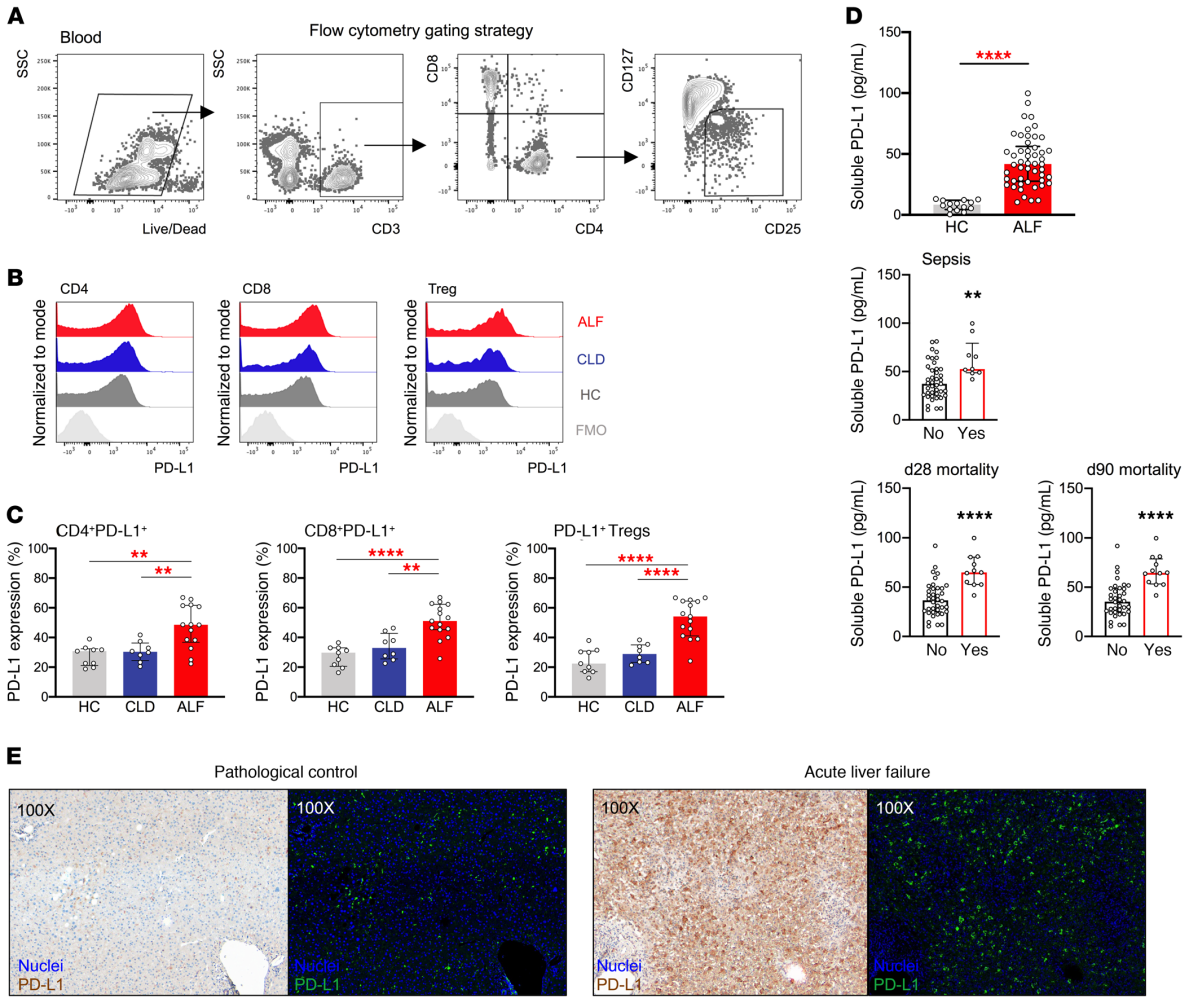
Lymphocyte PD-L1 expression and sPD-L1 plasma levels are increased in patients with ALF. Phenotypic characterization of lymphocytes was performed by flow cytometry in PBMCs from HCs (*n* = 9) and patients with CLD (*n* = 8) or ALF (*n* = 15). (**A**) Representative flow cytometric gating strategy used to identify CD4^+^ T cells, CD8^+^ T cells, and Tregs. (**B** and **C**) Representative histograms and data showing PD-L1 expression (percentage) in CD4^+^ T cells, CD8^+^ T cells, and Tregs. (**D**) Dot plots show plasma sPD-L1 levels as determined by ELISA in HCs (*n* = 8) and patients with ALF (*n* = 50) and plasma sPD-L1 levels in patients with ALF based on development of sepsis (no: *n* = 41; yes: *n* = 9), day-28 mortality (no: *n* = 38; yes: *n* = 11), or day-90 mortality (no: *n* = 36; yes: *n* = 12). (**E**) Representative IHC images of PD-L1 staining in pathological control and APAP-induced ALF liver tissues analyzed using Nuance multispectral imaging technology. Left panels: RGB images show nuclei (blue) and PD-L1 (brown) staining. Original magnification, ×100. Right panels: Pseudofluorescence images show nuclei (blue) and PD-L1 (green) staining. Original magnification, ×100. Data are presented as the median with the IQR. **P < 0.01 and ****P < 0.0001, by Mann-Whitney *U* test.

Having established that the PD-1/PD-L1 axis altered KC responses in murine acute liver injury, we sought to demonstrate that this translates to human disease and that the PD-1/PD-L1 pathway may be a potential therapeutic target. We aimed to assess PD-1 and PD-L1 expression in PBMCs from healthy controls (HCs) and from patients with stable CLD or ALF (Table 1). We identified peripheral lymphocyte subsets by flow cytometry (ref. 7, Figure 7A, and Supplemental Figure 8A) and found no differential PD-1 expression by lymphocytes among the study groups (Supplemental Figure 8, A–D). However, PD-L1 expression by CD4^+^ T cells, CD8^+^ T cells, and Tregs was significantly increased in patients with ALF (Figure 7, B and C), in both patients with acetaminophen-induced ALF (AALF) and those with non–acetaminophen-induced ALF (NAALF) (Supplemental Figure 8B). In line with this, IHC showed increased expression of hepatic PD-L1 in ALF explant tissue compared with expression in pathological controls (Figure 7E and Supplemental Figure 8E).

**Figure 8:**
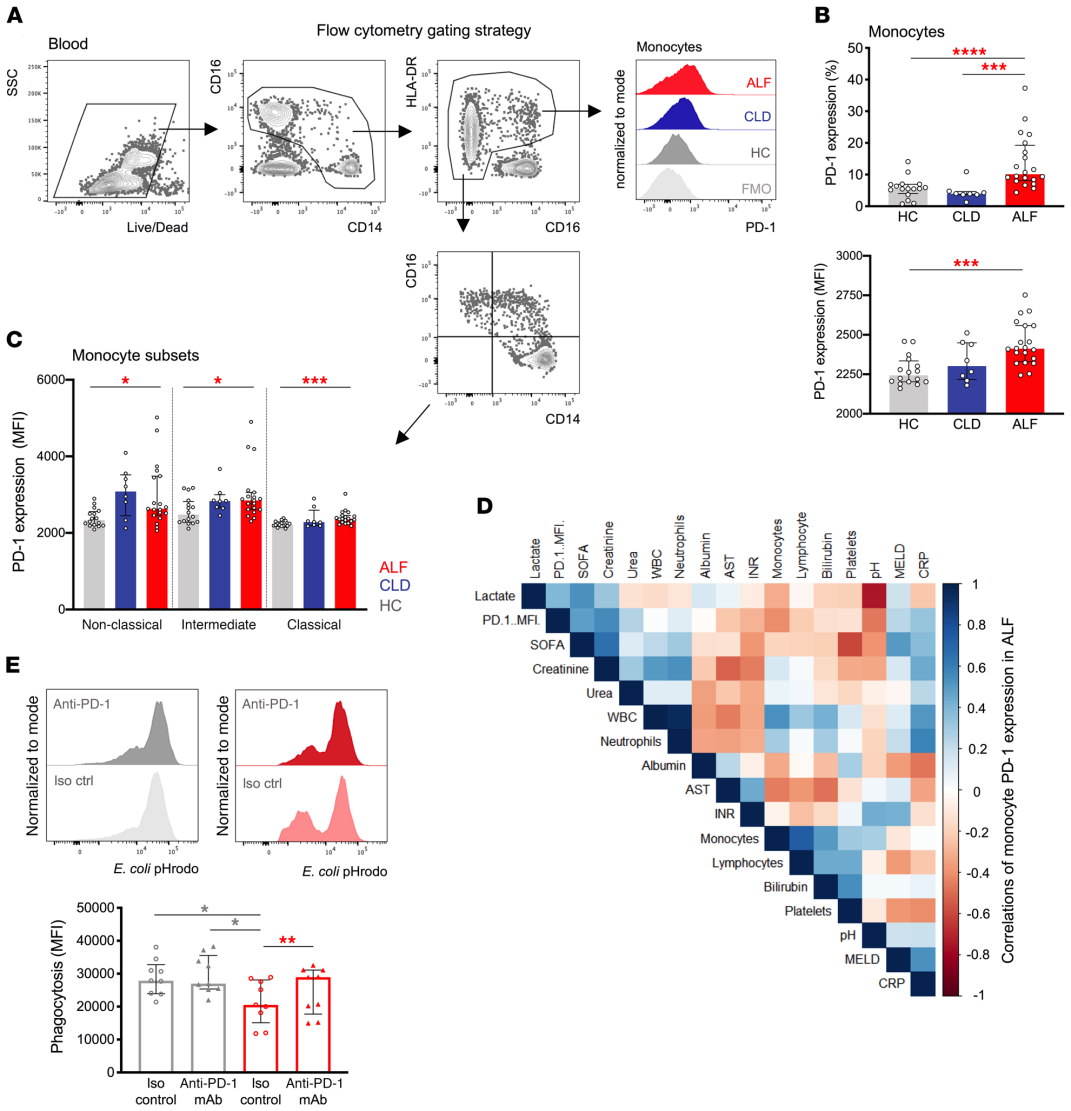
Monocyte PD-1 expression is increased in patients with ALF. Phenotypic characterization of monocytes was performed by flow cytometry in PBMCs from HCs (*n* = 16) and patients with CLD (*n* = 8) or ALF (*n* = 20). (**A**) Representative flow cytometric gating strategy used to identify monocytes and determine their PD-1 expression. Histograms show PD-1 expression in HCs and in patients with CLD or ALF. (**B** and **C**) PD-1 expression levels in (**B**) total monocytes and (**C**) monocyte subsets (classical, intermediate, and nonclassical). Statistical significance was determined by Mann-Whitney *U* test. (**D**) Matrix of correlation of monocyte PD-1 expression (MFI) with clinical scores and biochemical parameters for patients with ALF. INR, international normalized ratio; AST, aspartate aminotransferase; MELD, model for end-stage liver disease; WBC, white blood cell count. (**E**) PBMCs from HCs and patients with ALF were cultured in the presence of 10% autologous plasma and treated with anti–PD-1 mAb (10 μg/mL) or an isotype-matched control (iso ctrl) (10 μg/mL) prior to *E*. *coli* pHrodo phagocytosis assay. Flow cytometric plots and analysis show *E*. *coli* pHrodo phagocytosis levels (MFI) in HCs and patients with ALF (*n* = 9 per group). Results are from 3 independent experiments. Data are presented as the median with the IQR. *P < 0.05, **P < 0.01, ***P < 0.001, and **** *P* < 0.0001, by Wilcoxon paired test (ALF group) and Mann-Whitney *U* test (HCs versus patients with ALF).

In addition to its major membrane-bound form, a soluble form of PD-L1 (sPD-L1) can be found in plasma and is increased in a few cancers, where it is negatively associated with survival and the response to immunotherapy, suggesting an inhibitory effect (32, 33). We detected increased plasma levels of sPD-L1 in patients with ALF compared with levels in HCs (Supplemental Table 1 and Figure 8D). sPD-L1 plasma levels in patients with ALF were positively correlated with the sequential organ failure assessment (SOFA) score, as well as with creatinine and lactate levels, and were negatively correlated with pH (Supplemental Figure 9D). Notably, plasma sPD-L1 levels were significantly higher in patients with ALF who developed sepsis or had a poor outcome (Figure 7D).

### Increased monocyte PD-1 expression in patients with ALF.

We identified peripheral monocytes by flow cytometry (ref. 6 and Figure 8A). Compared with HCs and patients with CLD, we found that patients with ALF had increased proportions of PD-1^+^ monocytes and PD-1 expression levels (Figure 8B). Of note, the percentage of PD-1^+^ monocytes was higher in patients with AALF than in those with NAALF (Supplemental Figure 9A). PD-1 was detected at high levels in intermediate monocytes (Figure 8C) and was coexpressed with activation/pro-restorative markers in patients with ALF (refs. 4–6 and Supplemental Figure 9C). Also, ALF monocytes had higher PD-L1 expression (Supplemental Figure 9B), mirroring our murine macrophage data (Figure 2, E and F). We consistently observed increased expression of hepatic PD-1 and PD-L1 in ALF explant tissue, with more pronounced expression in AALF tissue, compared with pathological controls (Figure 7E, Supplemental Figure 8E, and Supplemental Figure 10, A and B). Monocyte PD-1 expression correlated positively with SOFA scores and lactate levels and negatively with pH and peripheral monocyte numbers (Figure 8D). Interestingly, PD-1 expression was higher in patients with ALF who developed sepsis or had a poor outcome (Supplemental Figure 9E).

### PD-1 in vitro blockade improves monocyte bacterial uptake in human ALF.

Compared with HCs and patients with CLD, monocytes from patients with ALF showed impaired *E*. *coli* phagocytosis (ref. 8 and Supplemental Figure 9F). We found that *E*. *coli* uptake was lower in PD-1^+^ versus PD-1^–^ monocytes in patients with ALF and that monocyte PD-1 expression inversely correlated with the phagocytosis index (Supplemental Figure 9F). We therefore explored the effects of anti–PD-1 treatment on PBMCs from HCs and patients with ALF. We demonstrate that PD-1 in vitro blockade restored monocyte *E*. *coli* uptake in patients with ALF (Figure 8E) and increased LPS-stimulated proinflammatory cytokines (Supplemental Figure 9G). Together, our data suggest that monocyte PD-1 expression and plasma sPD-L1 levels may be used as prognostic biomarkers and that PD-1 blockade can restore monocyte innate responses in ALF.

## Discussion

Our study reports impaired KC bacterial clearance and systemic bacterial dissemination after *E*. *coli* infection in mice with acute liver injury. We describe a role of the PD-1/PD-L1 pathway in regulating KC inflammatory and antimicrobial responses. We demonstrate that the tolerogenic effects of this axis involve PD-1^+^ KCs and PD-L1^+^ lymphocyte subsets that may provide suppressive back signaling. Gene expression profiling of PD-1**^+^** KCs revealed an immune-suppressive profile typified by reduced antigen presentation, lymphocyte activation, and pathogen responses. Notably, PD-1 deficiency or PD-1 blockade in mice with liver injury improved KC bacterial clearance, reduced the tissue bacterial burden, and conferred protection from sepsis after infection.

The regulatory role of PD-1/PD-L1 signaling in T cell function and differentiation is well documented (18, 19), however, accumulating evidence has associated PD-1 expression by macrophages with immune suppression in infection, sepsis, and cancer (23–27). Here, in the context of sterile liver inflammation, we found that PD-1 expression by KCs was enhanced during the resolution phase. PD-1 expression can be induced on monocytes and macrophages by TLR ligands (e.g., LPS) and various cytokines (TNF-α, IL-1β, and IL-6; refs. 21, 22). Such inflammatory cues are highly present in the hepatic microenvironment after injury (3, 17, 34) and could therefore be responsible for PD-1 induction in KCs.

Recent studies showed that PD-1^+^ TAMs exhibit an M2-like expression (CD206^hi^MHC class II^lo^CD64^lo^) profile and phagocytose fewer tumor cells or OVA in colon and gastric cancer, respectively, compared with PD-1^neg^ TAMs (26, 35). Here, we provide an in-depth transcriptomic profile of PD-1^+^ KCs during the resolution of liver injury. Consistent with the above data (26, 35), we found that PD-1^+^ KCs exhibited M2-like polarization (e.g., *Arg1*, *Cd206*) and immune-suppressive properties characterized by a reduction in antigen presentation and lymphocyte costimulation-related molecules (e.g., *Btk*, *Cd64*, *Cd80*, *Cd86*). Furthermore, PD-1^+^ KCs showed reduced expression of TLR signaling factors (e.g., *Irf5*, *Irf7*), antimicrobial defense molecules, including enzymes (e.g., *Aoah*, *Mpeg1*), and inflammasome-related factors (e.g., *Nlrp3,* *Il1b,* *Il18*). In addition, PD-1^+^ KCs have lower expression of *Cybb* and *Ncf2* genes that encode the membrane protein gp91phox and cytosolic subunit p67-phox of the NADPH oxidase complex, respectively. This complex is crucial for the production of ROS in phagocytes, and its reduction implies an inability of PD-1^+^ cells to kill the ingested pathogens. This is consistent with our data showing impaired KC bacterial killing during the resolution of injury. Altogether, our study demonstrates that PD-1 expression by KCs is associated with an immune-suppressive profile and reduced antimicrobial functions.

PD-1/PD-L1 signaling is crucial in regulating T cell responses (18, 19). We found that hepatic PD-L1^+^ lymphocyte subsets were expanded during the resolution of liver injury in mice. Likewise, in humans, we detected increased expression of peripheral lymphocyte PD-L1, hepatic PD-L1, and plasma sPD-L1 in patients with ALF. Reports have shown that PD-L1 in T cells can be induced in response to antigen presentation and sterile inflammatory cues (e.g., IFN-γ, IL-4), whereas PD-L1^+^ T cells can lead to tumor-promoting tolerance (28, 36). PD-L1^+^ T cells suppress neighboring effector T cells via the PD-L1/PD-1 axis and promote STAT6-dependent, M2-like tolerogenic macrophages in cancer (28). These findings are functionally relevant to our model, as the increase in PD-L1^+^ lymphocytes and sPD-L1 after injury may exert suppressive effects on PD-1^+^ KCs. Further work, for example in coculture experiments, could examine the effects of lymphocytes on monocyte and KC function.

KCs are important for maintaining liver tolerance. During homeostasis, KC-associated antigen presentation induces CD4^+^ T cell liver arrest and expansion of IL-10^+^ Tregs, however, this immunological tolerance is abrogated in chronic liver injury and fibrosis (37). Of note, in vivo interactions of liver-patrolling NKT cells with KCs are observed during steady-state and after infection (38–40). Here, we found PD-L1^hi^ NKT cells and Tregs during resolution (39, 41) of sterile acute liver injury. We speculate that these PD-L1^hi^ subsets interfacing with PD-1^+^ KCs may exert suppressive effects on the latter via the PD-1/PD-L1 axis. Future studies will need to delineate the in vivo interactions of lymphocytes with KCs during homeostasis and liver inflammation and further examine the PD-1/PD-L1 pathway and its regulatory role in the liver.

We found impaired KC bacterial clearance after liver injury and reduced pathogen responses of PD-1^+^ KCs, in line with other studies that have associated PD-1 with macrophage suppression. For instance, PD-1^–/–^ mice are protected from septic peritonitis, displaying improved bacterial clearance and a less severe cytokine storm (23), whereas PD-1 or PD-L1 mAb blockade leads to similar results (42, 43). We consistently found that both PD-1^–/–^ mice and anti–PD-1 mAb–treated mice with liver injury had unimpaired KC bacterial clearance and were protected from sepsis after systemic infection. Importantly, in mouse models, PD-1 deficiency or PD-1 mAb blockade did not exaggerate liver injury or delay its resolution, suggesting that PD-1 therapy may be a safe strategy to prevent immune suppression after liver injury. Strauss et al. found that PD-1 in myeloid cells dampened antitumor immunity and demonstrated that myeloid cell PD-1 (PD-1^fl/flLysMcre^) ablation was as effective as global PD-1 (PD-1^–/–^) ablation in limiting tumor growth, and considerably more effective than lymphocyte PD-1 (PD-1^fl/flCD4cre^) ablation (27). Given this recent evidence, the lack of such experimental models should be acknowledged as a limitation in our study. Future research using mice with myeloid- or KC-specific PD-1 ablation should examine in more detail the role of PD-1 in regulating the function of liver macrophages.

Peripheral monocyte dysfunction and its contribution to infection susceptibility is documented in patients with ALF. For instance, monocytes exhibit a reduction in proinflammatory cytokines and bacterial uptake (4–6, 8). PD-1 expression by monocytes is reportedly increased in human chronic lymphocytic leukemia (25), HIV or active tuberculosis (22, 24), and sepsis (23), whereas PD-1/PD-L1 blockade restores innate responses in sepsis (23, 44, 45). In conjunction with our murine liver findings, we report that the PD-1/PD-L1 pathway may contribute to peripheral monocyte suppression in human ALF, as we detected increased systemic levels of monocyte PD-1, lymphocyte PD-L1, and sPD-L1. We consistently observed increased hepatic PD-1 and PD-L1 expression in ALF. Of note, monocyte PD-1 and sPD-L1 levels were markedly higher at hospital admission in patients who had sepsis and a poor outcome and therefore may serve as prognostic biomarkers. Future prospective studies, including longitudinal patient sampling, are warranted to assess the utility of these markers in predicting immune suppression and mortality in patients with liver injury.

Immune checkpoint inhibition has recently been considered a therapeutic strategy in CLD. Defects in adaptive and humoral immunity can be partially rescued in vitro by PD-1 blockade, as shown for T cells in alcohol-related liver disease (46) and for B cells in viral hepatitis B infection (47). The safety and efficacy of PD-1/PD-L1 mAb blockade for the treatment of hepatocellular carcinoma has also been assessed in clinical trials, with promising results (48–50). However, immune checkpoint inhibition in myeloid cells and its contribution to the efficiency of such mAb-based therapies remain unexplored. Furthermore, patients with liver cirrhosis have a high risk of infection and mortality (51). Bacterial infections occur frequently in patients with cirrhosis (32%–34%, refs. 51, 52) and are the main precipitant for hepatic acute decompensation (AD), with or without organ failure (acute-on-chronic liver failure [ACLF]) (53, 54). Like ALF (3), cirrhosis and ACLF are associated with inflammation and, as disease progresses, immune dysfunction that contribute to increased susceptibility to infection (5, 8, 55–57). It would be important to further investigate the significance of the PD-1/PD-L1 pathway in cirrhosis, AD, and ACLF and its contribution to systemic and hepatic immune suppression.

In conclusion, we describe a crucial role of the PD-1/PD-L1 axis in regulating KC and monocyte inflammatory and antimicrobial responses following acute liver injury. Our findings suggest that PD-1–targeted immunotherapy may be a strategy to prevent innate immune suppression in ALF.

## Methods

### Mice.

C57BL/6 WT mice were purchased from Charles River Laboratories, and PD-1^–/–^ (B6.Cg-*Pdcd1^tm1.1Shr^*/J) mice were purchased from The Jackson laboratory. Mice were housed and bred at the Imperial College London animal facility under specific pathogen–free conditions. For induction of sterile acute liver injury, male mice (aged 8–12 weeks) were fasted overnight and received an intraperitoneal injection of 250 mg/kg APAP (MilliporeSigma) diluted in saline. WT mice received an intraperitoneal injection of either 200 μg anti–PD-1 mAb (clone RMP1-14, BioXCell) or 200 μg IgG2a control (clone 2A3, BioXCell) diluted in saline.

### Patients.

Patients with ALF (*n* = 50) (Table 1 and Supplemental Table 1) were recruited to the study within 24 hours of their admission to the Liver Intensive Therapy Unit (LITU) of King’s College Hospital. Inpatients with CLD (*n* = 8) and HC volunteers (*n* = 16) (Table 1) were recruited and served as pathological controls and HCs, respectively. Liver tissue was obtained from patients with ALF (*n* = 4) undergoing orthotopic liver transplantation (OLT), and pathological control tissue was derived from hepatic resection margins of colorectal malignancies (*n* = 3). Exclusion criteria included age younger than 18 years, neoplasia, and immunosuppressive therapy. Biochemical parameters were determined on a hematological analyzer (Advia 2120, Siemens).

### Intravital microscopy.

Intravital imaging of mice was performed by confocal microscopy. For imaging of bacterial capture, hepatic macrophages and endothelial cells were visualized by intravenous injection of anti–F4/80-BV421 (5 μg/mouse, BM8, BioLegend) and anti–CD31-phycoerythrin (anti–CD31-PE) (5 μg/mouse, MEC13.3, BioLegend) antibodies. Mice were anesthetized by intraperitoneal injection of ketamine/medetomidine (125/1 mg/kg). After a midabdominal incision was made, the mouse was placed in a right lateral position, and the largest liver lobe was externalized on a coverslip attached to the microscope stage. The liver was covered with saline-moistened lab wipes to restrict movement, and the microscope heating chamber was kept at 37°C to maintain mouse body temperature. The liver was then imaged with an inverted Leica TCS SP5 confocal microscope with the Leica DM6000 Confocal Fixed Stage system. Bacteria that remained associated with KCs for more than 2 minutes were considered captured. Time-lapse images were recorded for up to 20 minutes after infection. A total of 5–6 field of view (FOV) images were recorded for each mouse under a ×20 microscope objective, and their average was considered 1 FOV. Videos and images were processed and analyzed with Imaris 8 software (Bitplane).

### Bacterial challenge.

*E. coli* GFP (25922GFP, American Type Culture Collection [ATCC]) bacteria were cultivated in Tryptic Soy Agar/Broth (MilliporeSigma). For flow cytometry, mice received an intravenous dose of 5 × 10^6^ *E*. *coli* GFP (per 20 g BW, ref. 10). Five minutes after *E*. *coli* injection, 10 μL blood was obtained, diluted (1:40) in PBS, and analyzed on a BD LSRFortessa Cell Analyzer (BD Biosciences). The number of GFP^+^ events was used to calculate free *E*. *coli* in the circulation using 123count eBeads Counting Beads (Invitrogen, Thermo Fisher Scientific). For intravital imaging of *E*. *coli* capture in the liver, mice received an intravenous dose of 5 × 10^7^ *E*. *coli* GFP (per 20 g BW, ref. 10) and were imaged under confocal microscopy for 20 minutes after infection. For sepsis studies, mice received an intravenous nonlethal dose of 5 × 10^7^ *E*. *coli* GFP bacteria (per 20 g BW). A murine sepsis scoring system (29) was used to evaluate disease severity. Animal health monitoring was conducted by 2 investigators every 2 hours after infection (for 12 hours), with evaluation of appearance, level of consciousness, activity, response to stimuli, eyes, and respiration rate and quality (29). For evaluation of tissue bacterial burden, organs were harvested, weighed, and homogenized in 1 mL PBS. Tissue suspensions were plated onto agar plates at serial dilutions, and CFU were counted after an 18-hour incubation at 37°C.

### Bacterial killing assay.

Bacterial killing of KCs was measured in cell lysate fractions (58). Briefly, sorted KCs (20,000 cells) from control and APAP-treated (72 h) mice were incubated in 48-well, flat-bottomed plates (37°C, 5% CO_2_) with *E*. *coli* GFP (ATCC 25922GFP) at a 1:100 ratio in DMEM (Gibco, Thermo Fisher Scientific) supplemented with 10% autologous plasma. After a 60-minute incubation, the plates were put on ice, and supernatants were aspirated. Cell pellets were next lysed in distilled water (pH 11), lysate fractions were plated onto agar plates at serial dilutions, and CFU were counted after an 18-hour incubation at 37°C.

### Flow cytometry.

Mouse hepatic nonparenchymal cells were isolated (14) for phenotypic characterization of myeloid and lymphoid cell subsets by flow cytometry. Human PBMCs were isolated as previously described (6). Blood monocytes and lymphoid cell subsets were phenotypically characterized by flow cytometry. Details on the Abs used for flow cytometric staining are provided in the supplemental material. Data acquisition was performed on a BD LSRFortessa Cell Analyzer (BD Biosciences). Flow cytometric data analysis was performed using FlowJo, version 10.

### NanoString nCounter.

KCs from APAP-treated WT mice (72 h) were FACS-sorted using a FACSAria Fusion Analyzer (BD Biosciences). mRNA expression was analyzed with the NanoString nCounter Mouse Myeloid Innate Immunity Panel (734 immunology-related and 20 housekeeping genes). Data were analyzed using nCounter Advanced Analysis software (NanoString Technologies). Normalization (geNorm) and *P* value adjustment (Benjamini-Hochberg) were applied, as detailed in the Supplemental Methods. Statistically significant results were considered at a *P* value of less than 0.05 and a fold change of 2.

For further technical information, see the supplemental materials.

### Statistics.

Data graph presentation and statistical analyses were performed using GraphPad Prism 8 (GraphPad Software). Data are presented as the mean ± SEM, unless stated otherwise. A Mann-Whitney *U* nonparametric test was used for statistical analysis of differences between 2 groups. A 1-way ANOVA with Dunnett’s or Tukey’s multiple-comparison test was used for analysis of differences between more than 2 groups. A 2-way ANOVA with Tukey’s multiple-comparison test was used for analysis of differences between more than 2 groups and with 2 different independent variables. A Spearman’s nonparametric correlation test was used for associations with clinical parameters. A *P* value of less than 0.05 was considered statistically significant.

### Study approval.

All animal experimental protocols were approved by the Imperial College London in accordance with UK Home Office regulations (PPL: P8999BD42). The human study was approved by the UK National Health Service (NHS) Health Research Authority (LREC: 12/LO/0167, 15/LO/0363). Assent was given by the patients’ nominated next of kin if the patients were unable to provide informed consent themselves.

## Author contributions

ET, CGA, and MRT conceptualized the study. ET, CLCG, and MAM performed mouse experiments. ET, CLCG, MAM, HCH, FMT, MKS, TO, HK, CB, SKM, RDG, and MJWM collected, analyzed, and interpreted data. ET, WK, LAP, KJW, and MRT wrote the original draft of the manuscript. ET, CLCG, MKS, MB, WK, MJWM, LAP, KJW, JAW, RDG, and MRT wrote and critically revised the manuscript. ET, CGA, and MRT acquired funding.

## Supplementary Material

Supplemental data

Supplemental Data Set 1

Supplemental Video 1

Supplemental Video 2

## Figures and Tables

**Table 1:**
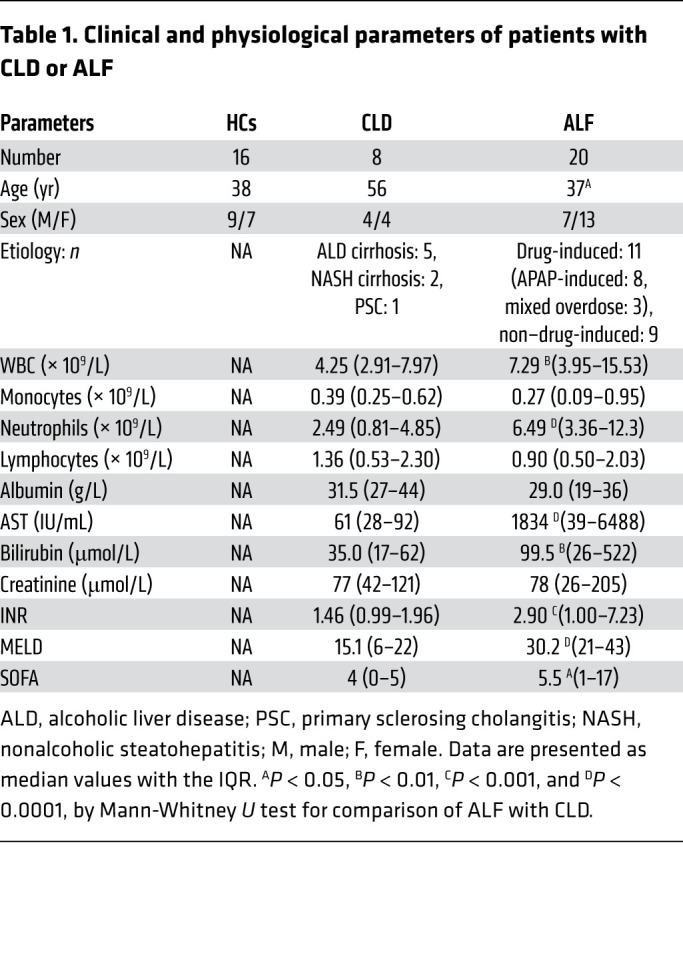
Clinical and physiological parameters of patients with CLD or ALF

## Version 1 12/15/2020

In-Press Preview

## Version 2 02/15/2021

Electronic publication
