# Supplementary material for: PD-1 blockade improves Kupffer cell bacterial clearance in acute liver injury
Source: J Clin Invest. 2021 Feb 15;131(4):e140196. doi: 10.1172/JCI140196 (PMC7880414; doi:10.1172/JCI140196)
Supplement: Supplemental data [file jci-131-140196-s066.pdf]

Supplemental Material

A. Supplemental Figures, Tables and Videos

Fig. S1: Kupffer cell *E. coli* capture, plasma ALT and liver PD-L1 mRNA levels in WT mice.

Fig. S2: PD-1+ and PD-L1+ liver macrophage and lymphocyte subsets in WT mice.

Fig. S3: Gene expression profiling of PD-1+ and PD-1- Kupffer cells after acute liver injury in WT mice.

Fig. S4: Indices of injury, H&E liver stains and blood immune cell subsets in WT and PD-1$^{-/-}$ mice.

Fig. S5: Liver myeloid and lymphoid subsets in WT and PD-1$^{-/-}$ mice.

Fig. S6: Plasma levels of inflammatory markers post *E. coli* infection in WT and PD-1$^{-/-}$ mice.

Fig. S7: Anti-PD-1 treatment restores Kupffer cell *E. coli* pHrodo uptake after acute liver injury.

Fig. S8: Peripheral lymphocyte and hepatic PD-L1 expression are increased in human acute liver failure.

Fig. S9: Phenotypic and functional characterization of peripheral monocytes in human acute liver failure.

Fig. S10: Hepatic PD-1 expression is increased in human acute liver failure.

Video S1. Intravital imaging of Kupffer cell bacterial capture in the liver.

Video S2. 3D reconstitution of liver intravital imaging with increasing transparency showing bacteria inside Kupffer cells.

Table S1: Clinical and physiological parameters of patients with acute liver failure (ALF) - soluble PD-L1 levels.

B. Supplemental Methods

Table S2. Primers used for quantitative reverse transcription PCR (RT-qPCR) in WT and PD-1$^{-/-}$ mice.

Table S3. Anti-mouse monoclonal antibodies used for phenotypic characterization of mouse blood immune cells.

Table S4. Anti-mouse monoclonal antibodies used for phenotypic characterization of mouse liver myeloid cells.

Table S5. Anti-mouse monoclonal antibodies used for phenotypic characterization of mouse liver lymphoid cells.

Table S6. Liver tissue weights of WT and PD-1$^{-/-}$ mice used for flow cytometry experiments.

Table S7. Liver tissue weights of WT and PD-1$^{-/-}$ mice used for bacterial burden (CFU) experiments.

Table S8. Anti-human monoclonal antibodies used for phenotypic characterization of human blood monocytes.

Table S9. Anti-human monoclonal antibodies used for phenotypic characterization of human blood.

A. Supplemental Figures, Tables and Videos

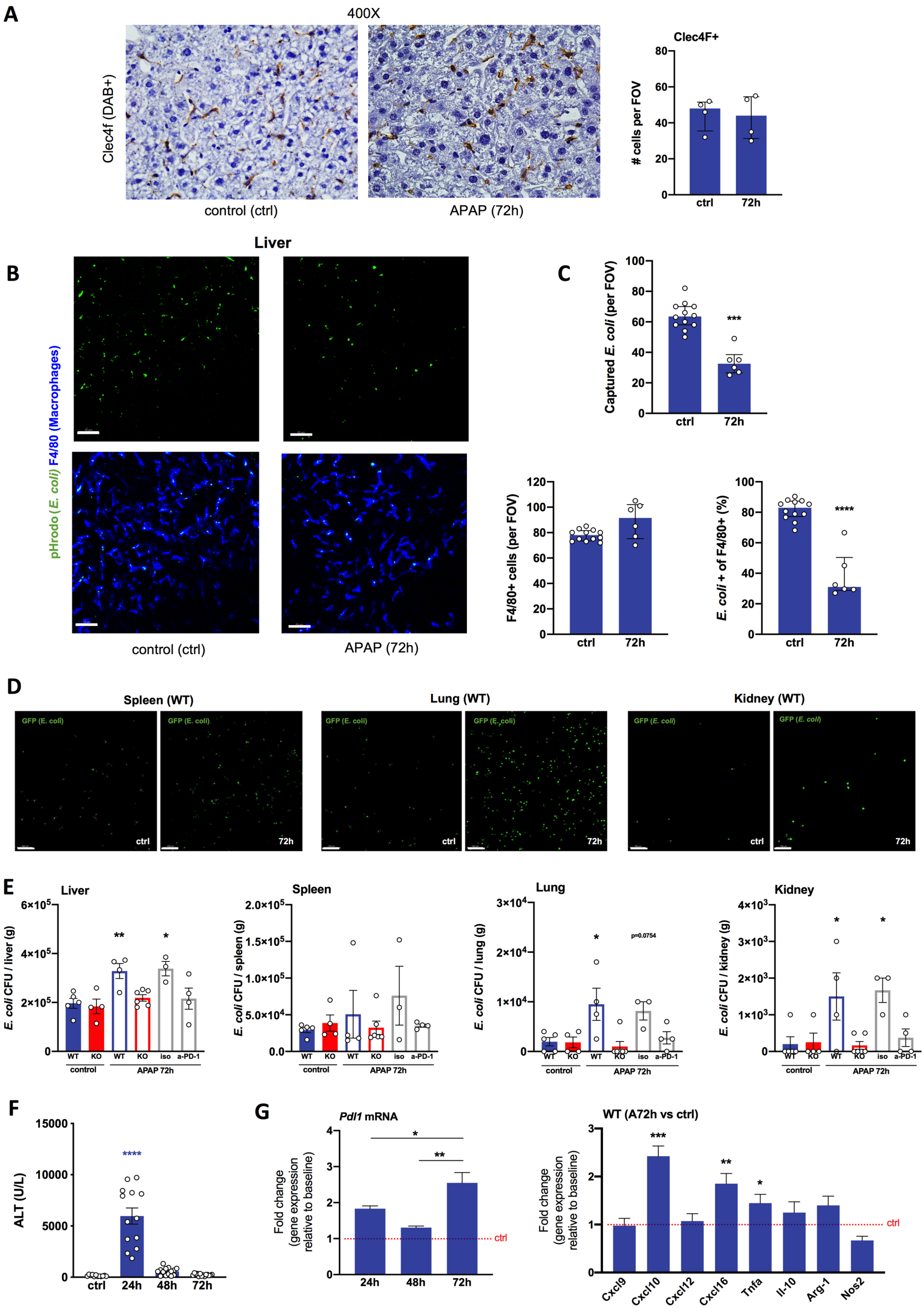

Fig. S1. Kupffer cell *E. coli* capture, plasma ALT and liver PD-L1 mRNA levels in WT mice. Baseline (control) and APAP-treated (24h, 48h, 72h) wild-type (WT) mice were studied. **(A)** Representative images of Clec4f liver tissue staining (400X magnification) and counts of Clec4f+ positive cells per field-of-view (FOV) (n=4 per group). **(B)** WT mice were intravenously challenged with *E. coli* pHrodo. Representative liver intravital images from baseline (control) and APAP-treated mice at 20 min post challenge. Macrophages were stained with fluorescently labelled anti-F4/80 antibody. Scale bars: 50 µm. **(C)** Intravital imaging analysis showing the amount of captured *E. coli* pHrodo, F4/80+ cells and percentage of F4/80+ *E. coli*+ cells per FOV; baseline (control, n=3) and APAP-treated (72h, n=2) mice; each dot shows counts per single FOV. Results are from two independent experiments; Mann-Whitney test used. **(D-E)** Representative confocal images (scale bars: 100 µm) and CFU number showing tissue bacterial burden at 20 min post *E. coli* GFP infection in different groups of mice. Groups included: (i) baseline (control) WT mice (n=5), (ii) baseline (control) PD-1$^{-/-}$ mice (n=4), (iii) APAP-treated WT mice (n=4), (iv) APAP-treated PD-1$^{-/-}$mice (n=6), (v) APAP-treated WT mice dosed (48h post-APAP) with isotype control (n=3), (vi) APAP-treated WT mice dosed (48h post-APAP) with anti-PD-1 monoclonal antibody (n=4). Each symbol represents an individual mouse. Results are from three independent experiments. (**F)** Data show plasma alanine transaminase (ALT) levels. **(G)** (Left) Liver tissue *Pdl1* relative gene expression (vs baseline) in WT mice (n=4 per time-point); (Right) Relative gene expression (vs baseline) in liver tissue in APAP-treated (72h) WT mice (n=4-8 each). CFU: colony forming unit. GFP: green fluorescent protein. * P< 0.05, ** P< 0.01, *** P< .001.

Macrophage subsets during murine APAP-induced acute liver injury

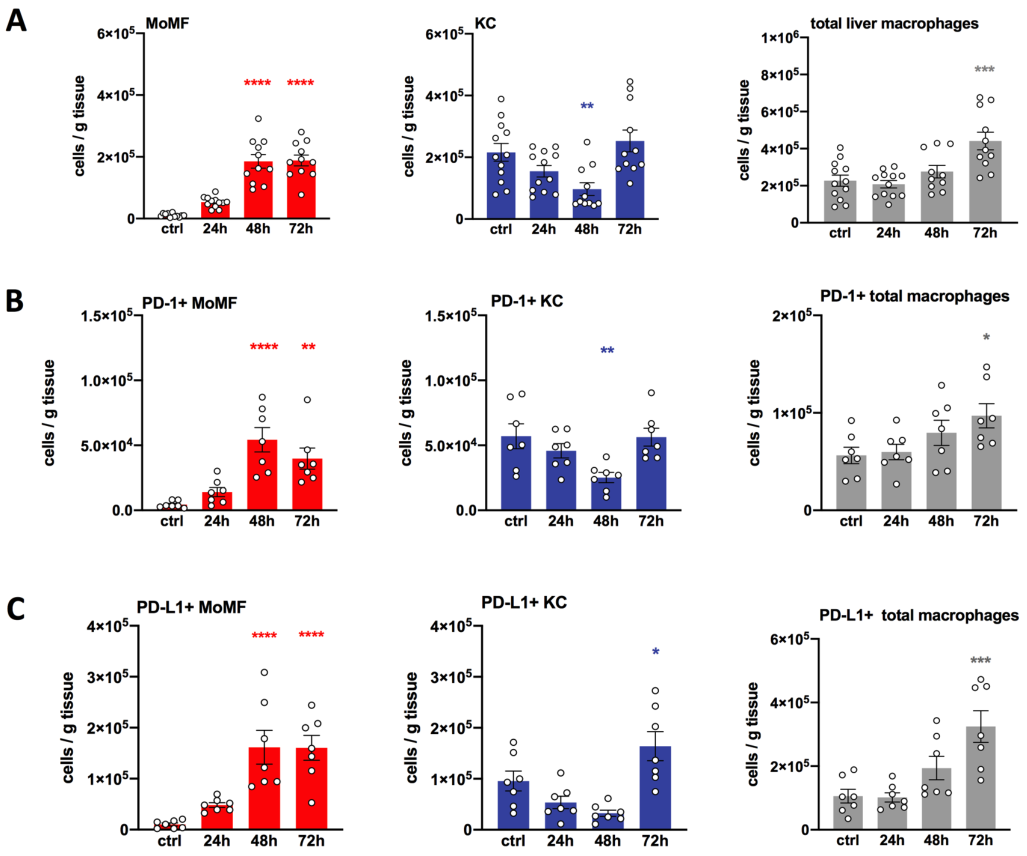

Lymphocyte subsets during murine APAP-induced acute liver injury

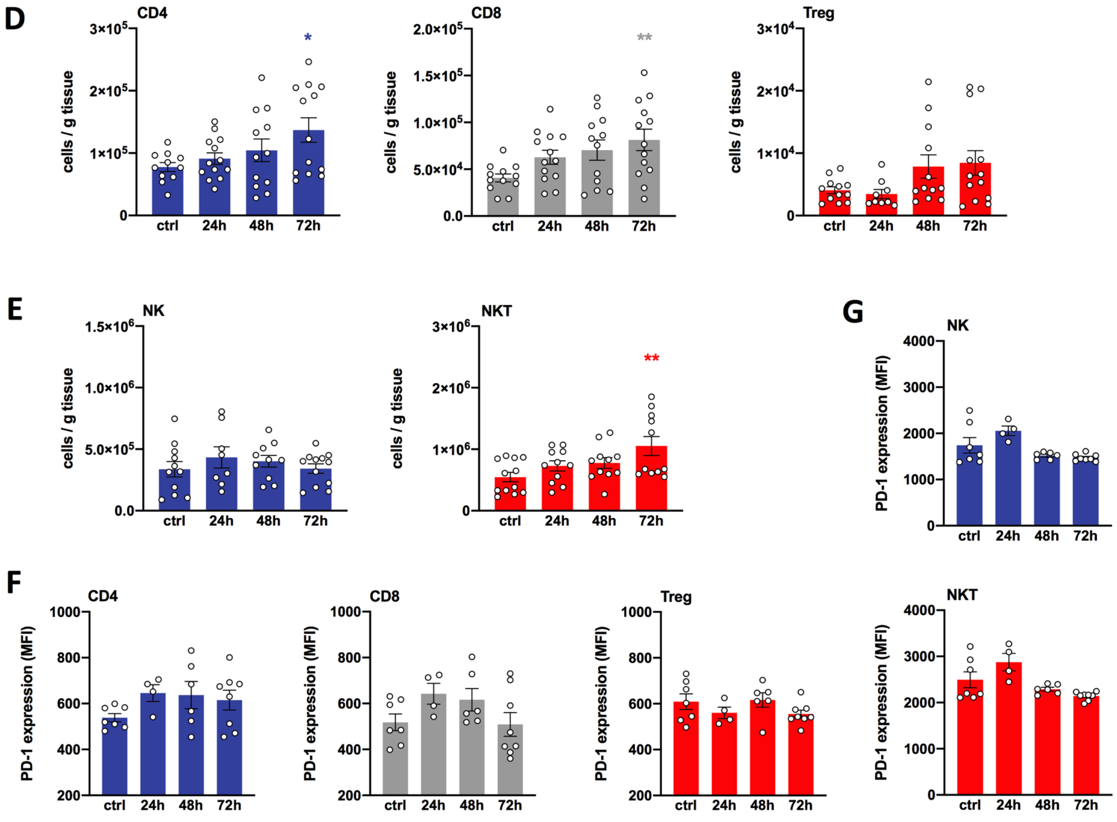

Fig. S2. PD-1+ and PD-L1+ liver macrophage and lymphocyte subsets in WT mice. Hepatic nonparenchymal cells were isolated from the livers of baseline (control) and APAP-treated (24h, 48h, 72h) WT mice. Phenotypic characterization of CD45+ leukocytes was performed using flow cytometry. **(A)** Number of monocyte-derived macrophages (MoMF, red), Kupffer cells (KC, blue) and total liver macrophages (grey) (n=10-12 per time point). **(B)** Number of PD-1+ MoMF, KC and total liver macrophages (n=7 per time point). (A-B) Results are from two independent experiments. **(C)** Number of PD-L1+ MoMF, KC and total liver macrophages (n=7 per time point). Results are from two independent experiments. **(D)** Number of CD4 (blue), CD8 (grey) and Treg (red) cells (n=12 per time point). **(E)** Number of NK (blue) and NKT (red) cells (n=8-12 per time point). (D-E) Results are from three independent experiments. **(F-G)** Data show PD-1 expression of CD4, CD8, Treg, NK and NKT cells (n=4-8 per time point). Results are from two independent experiments. Each symbol represents an individual mouse in all graphs. Data presented as mean ± SEM. One-way ANOVA test used (compared to control). MFI: mean fluorescence intensity. * $P< 0.05$, ** $P< 0.01$, *** $P< .001$, **** $P< .0001$.

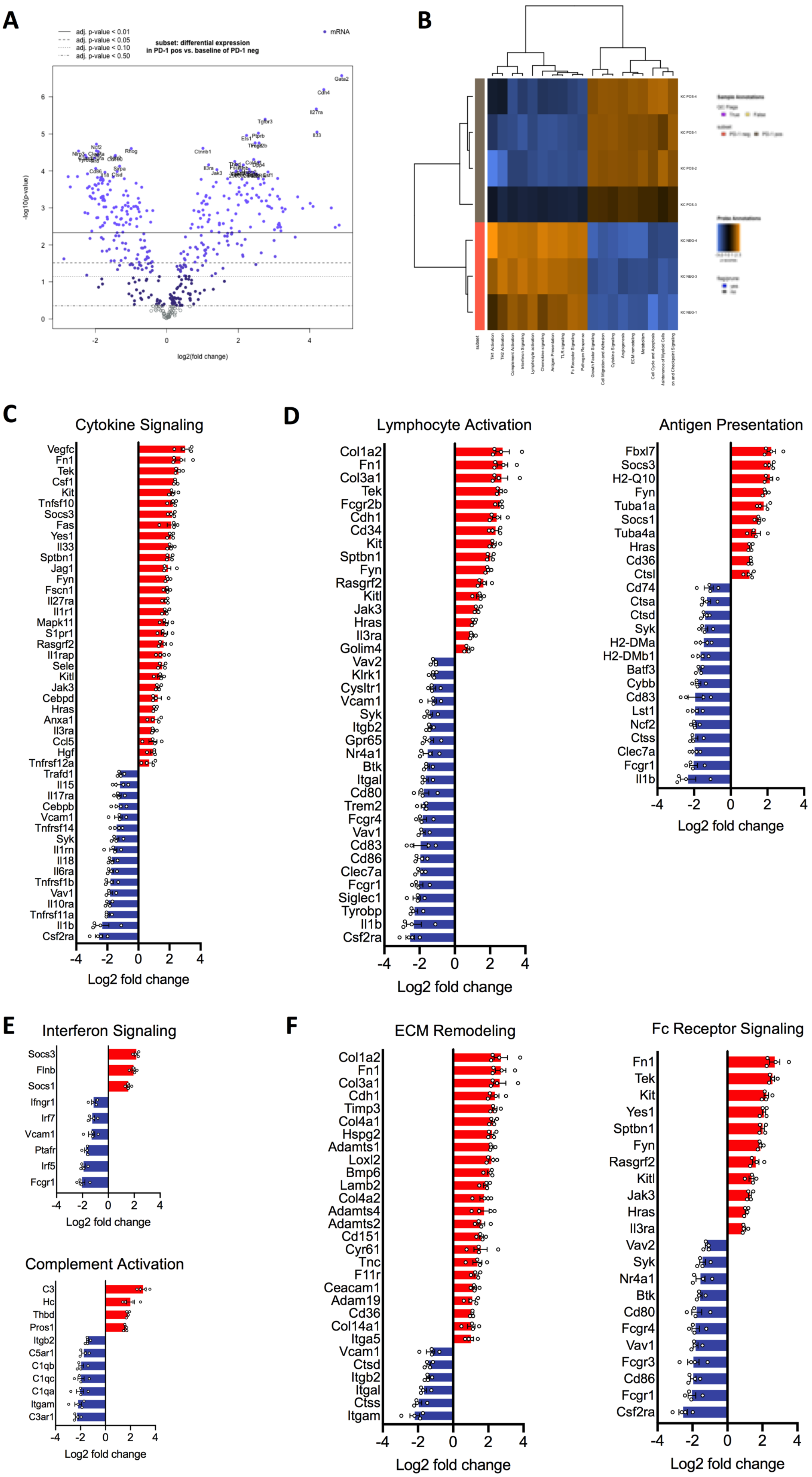
A
adj. p-value < 0.01
adj. p-value < 0.05
adj. p-value < 0.10
adj. p-value < 0.50
subset: differential expression
in PD-1 pos vs. baseline of PD-1 neg
mRNA
-log10(p-value)
log2(fold change)
Gata2
Cdh4
Il27ra
Tgfbr3
Il33
Ctnnb1
Rhog
Jak3
B
C
Cytokine Signaling
Vegfc
Fn1
Tek
Csf1
Kit
Tnfsf10
Socs3
Fas
Yes1
Il33
Sptbn1
Jag1
Fyn
Fscn1
Il27ra
Il1r1
Mapk11
S1pr1
Rasgrf2
Il1rap
Sele
Kitl
Jak3
Cebpd
Hras
Anxa1
Il3ra
Ccl5
Hgf
Tnfrsf12a
Trafd1
Il15
Il17ra
Cebpb
Vcam1
Tnfrsf14
Syk
Il1rn
Il18
Il6ra
Tnfrsf1b
Vav1
Il10ra
Tnfrsf11a
Il1b
Csf2ra
Log2 fold change
D
Lymphocyte Activation
Col1a2
Fn1
Col3a1
Tek
Fcgr2b
Cdh1
Cd34
Kit
Sptbn1
Fyn
Rasgrf2
Kitl
Jak3
Hras
Il3ra
Golim4
Vav2
Klrk1
Cysltr1
Vcam1
Syk
Itgb2
Gpr65
Nr4a1
Btk
Itgal
Cd80
Trem2
Fcgr4
Vav1
Cd83
Cd86
Clec7a
Fcgr1
Siglec1
Tyrobp
Il1b
Csf2ra
Log2 fold change
Antigen Presentation
Fbxl7
Socs3
H2-Q10
Fyn
Tuba1a
Socs1
Tuba4a
Hras
Cd36
Ctsl
Cd74
Ctsa
Ctsd
Syk
H2-DMa
H2-DMb1
Batf3
Cybb
Cd83
Lst1
Ncf2
Ctss
Clec7a
Fcgr1
Il1b
Log2 fold change
E
Interferon Signaling
Socs3
Ifnb
Socs1
Ifngr1
Irf7
Vcam1
Ptafr
Irf5
Fcgr1
Log2 fold change
Complement Activation
C3
Hc
Thbd
Pros1
Itgb2
C5ar1
C1qb
C1qc
C1qa
Itgam
C3ar1
Log2 fold change
F
ECM Remodeling
Col1a2
Fn1
Col3a1
Cdh1
Timp3
Col4a1
Hspg2
Adamts1
Loxl2
Bmp6
Lamb2
Col4a2
Adamts4
Adamts2
Cd151
Cyr61
Tnc
F11r
Ceacam1
Adam19
Cd36
Col14a1
Itga5
Vcam1
Ctsd
Itgb2
Itgal
Ctss
Itgam
Log2 fold change
Fc Receptor Signaling
Fn1
Tek
Kit
Yes1
Sptbn1
Fyn
Rasgrf2
Kitl
Jak3
Hras
Il3ra
Vav2
Syk
Nr4a1
Btk
Cd80
Fcgr4
Vav1
Fcgr3
Cd86
Fcgr1
Csf2ra
Log2 fold change

Fig. S3. Gene expression profiling of PD-1+ and PD-1- Kupffer cells following acute liver injury in WT mice. Kupffer cell subsets were sorted from livers of APAP-treated (72h) WT mice (n=4) using flow cytometry. PD-1+ and PD-1- Kupffer cell lysates were assessed for mRNA expression using a NanoString nCounter® Mouse Myeloid Innate Immunity Panel. **(A)** Volcano plot shows the top 20 differentially expressed genes between the subsets (based on nCounter® Advanced Analysis). **(B)** Heatmap showing the pathway score per individual sample (based on nCounter® Advanced Analysis). **(C-F)** Data show log2 fold-change of normalized linear count data (PD-1+ subset, n=4) of significantly differentially expressed genes (based on nCounter® Advanced Analysis) in various pathways. P-value adjustment (Benjamini-Hochberg) was applied. Data presented as mean values ± SEM. Statistically significant results were considered with $p<0.05$ and two-fold linear change.

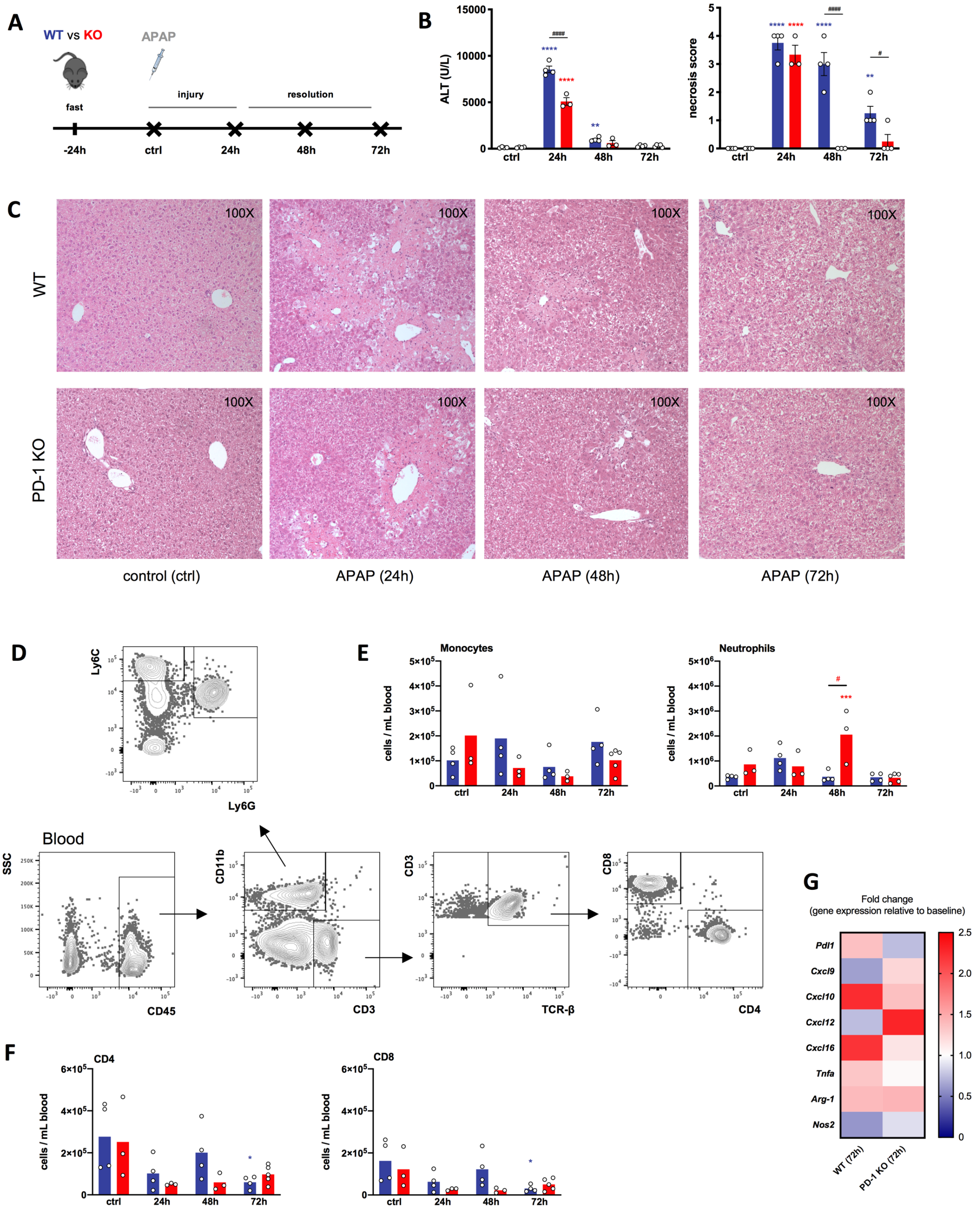
A
WT vs KO
APAP
fast
injury
resolution
-24h
ctrl
24h
48h
72h
B
ALT (U/L)
necrosis score
C
WT
PD-1 KO
100X
control (ctrl)
APAP (24h)
APAP (48h)
APAP (72h)
D
Ly6C
Ly6G
E
Monocytes
Neutrophils
cells / mL blood
Blood
SSC
CD45
CD11b
CD3
TCR-β
CD8
CD4
G
Fold change
(gene expression relative to baseline)
Pdl1
Cxcl9
Cxcl10
Cxcl12
Cxcl16
Tnfa
Arg-1
Nos2
WT (72h)
PD-1 KO (72h)
F
CD4
CD8

Fig. S4. Indices of injury, H&E liver stains and blood immune cell subsets in WT and PD-1$^{-/-}$ mice. Baseline (control) and APAP (24h, 48h or 72h) treated WT and PD-1$^{-/-}$ mice were studied. Each symbol in graphs represents an individual mouse. **(A)** Schematic of experimental APAP-induced acute liver injury in mice. **(B)** Data show plasma alanine transaminase (ALT) levels and hepatic necrosis score (n=3-4 per group per time point). Necrosis score: (0=none), (1=perivenular swelling), (2=perivenular necrosis), (3=bridging necrosis), (4=extensive bridging necrosis), (5=peri-lobular necrosis). (**C**) Representative H&E liver staining images (100X magnification). **(D)** Phenotypic characterization of blood CD45+ leukocytes was performed by flow cytometry. Representative flow cytometry gating strategy used to identify monocytes, neutrophils, CD4+ and CD8+ T cells is shown. **(E)** Number of blood monocytes and neutrophils (n=3-4 per group per time point). **(F)** Number of blood CD4 and CD8 T cells (n=3-4 per group per time point). Results are from two independent experiments. Two-way ANOVA test used in (B, E, F). **(G)** APAP-treated (72h) liver relative gene expression (vs baseline) as measured by RT-qPCR; Mann-Whitney test used. * $P< 0.05$, ** $P< 0.01$, *** $P< 0.001$, **** $P< 0.0001$.

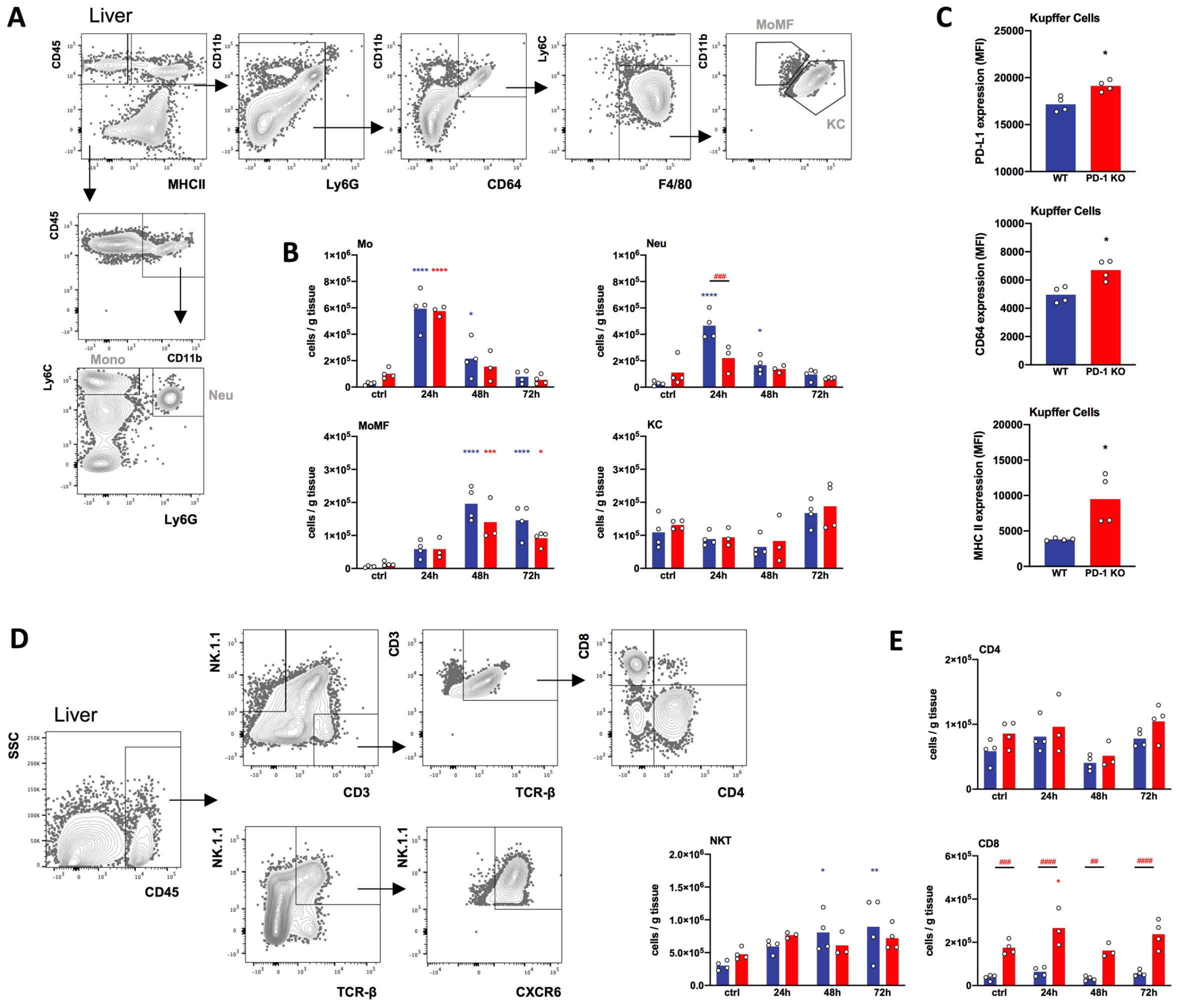

Fig. S5. Liver myeloid and lymphoid subsets in WT and PD-1$^{-/-}$ mice. Hepatic nonparenchymal cells were isolated from livers of baseline (control) and APAP-treated WT and PD-1$^{-/-}$ mice. Phenotypic characterization of liver CD45+ leukocytes was performed by flow cytometry. **(A)** Representative flow cytometry gating strategy used to identify monocytes, neutrophils, Kupffer cells (KC) and monocyte-derived macrophages (MoMF). **(B)** Number of monocytes, neutrophils, MoMF and KC (n=3-4 per group per time point). **(C)** CD64, MHCII and PD-L1 expression levels of Kupffer cells in APAP-treated (72h) mice (n=4 per group). **(D)** Representative flow cytometry gating strategy used to identify CD4+ T cells, CD8+ T cells and NKT cells. **(E)** Number of CD4, CD8 and NKT cells (n=3-4 per group per time point). Results are from two independent experiments. Two-way ANOVA test used in (B, E). Mann-Whitney test used in (C). * P< 0.05, ** P< 0.01, *** P< 0.001, **** P< 0.0001.

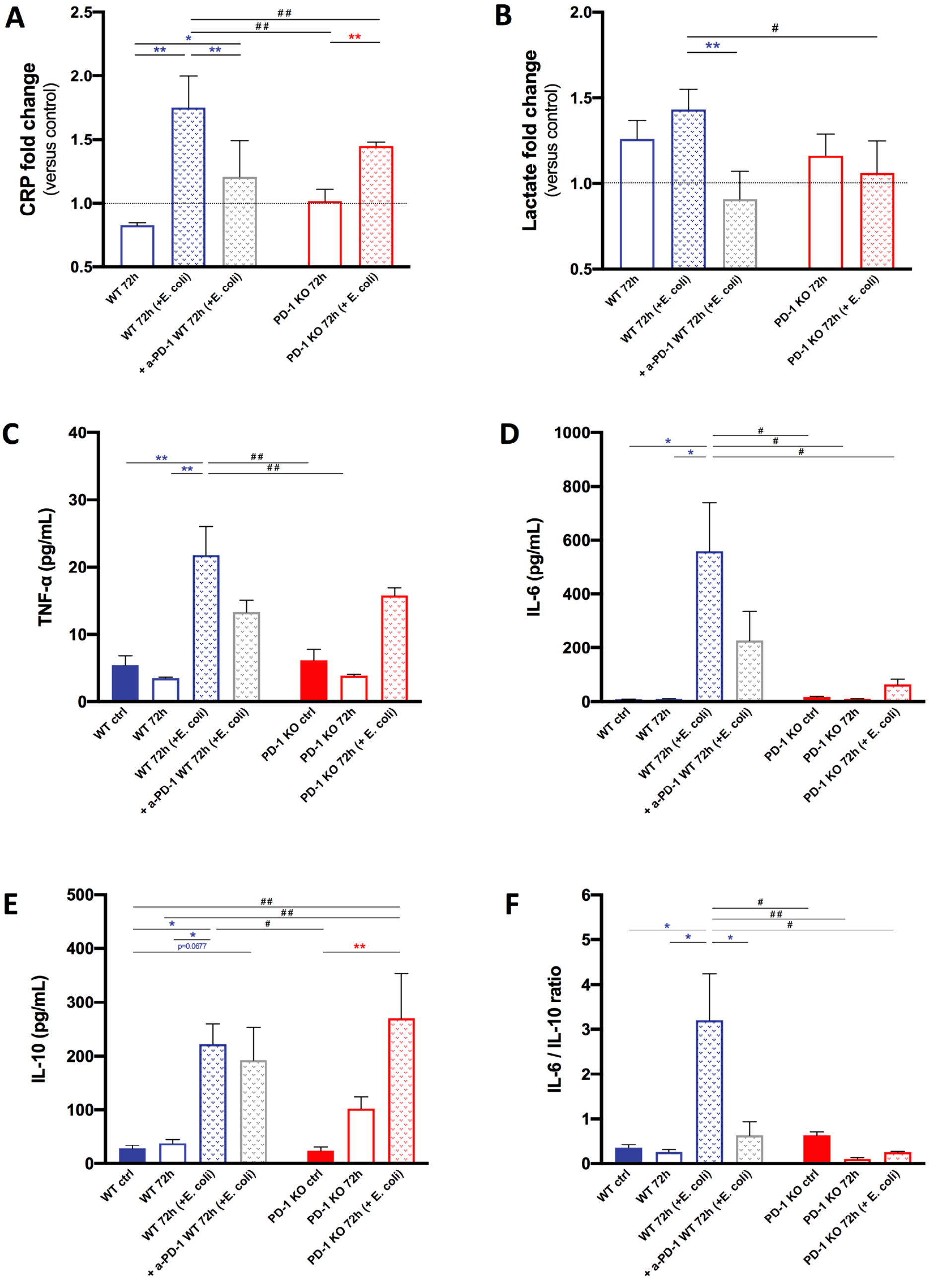

Fig. S6: Plasma levels of inflammatory markers post *E. coli* infection in WT and PD-1$^{-/-}$ mice. Systemic levels of inflammatory markers were measured in plasma derived from control (ctrl) or APAP-treated (72h) wild-type (WT) and PD-1-deficient (PD-1 KO) mice that were, or not, infected with *E. coli* for total 24 hours. (**A**) Data show C-reactive protein (CRP) levels in different treatment groups. CRP levels are expressed as fold-change relative to group (WT and PD-1 KO) matched baseline (ctrl) levels. **(B)** Data show lactate levels in different treatment groups. Lactate levels are expressed as fold-change relative to group (WT and PD-1 KO) matched baseline (ctrl) levels. **(C-E)** Data show inflammatory (TNF-α, IL-6) and anti-inflammatory (IL-10) cytokine levels (pg/mL) in different treatment groups. (**F**) Data show the IL-6/IL-10 ratio in same (C-E) groups. Treatment groups included: WT ctrl (n=4); WT APAP 72h (n=4); WT APAP 72h with *E. coli* infection (n= 6-8); WT anti-PD-1 treated APAP 72h with *E. coli* infection (n=4); PD-1 KO ctrl (n=4); PD-1 KO APAP 72h (n= 4); PD-1 KO APAP 72h with *E. coli* infection (n= 4-5). Mann-Whitney test was used in (A-B) and one-way ANOVA with Tukey's multiple comparisons test was used in (C-F). CRP: C-reactive protein. IL: interleukin. TNF-α: tumor necrosis factor-alpha. * P< 0.05, ** P< 0.01, # P< 0.05, ## P< 0.01.

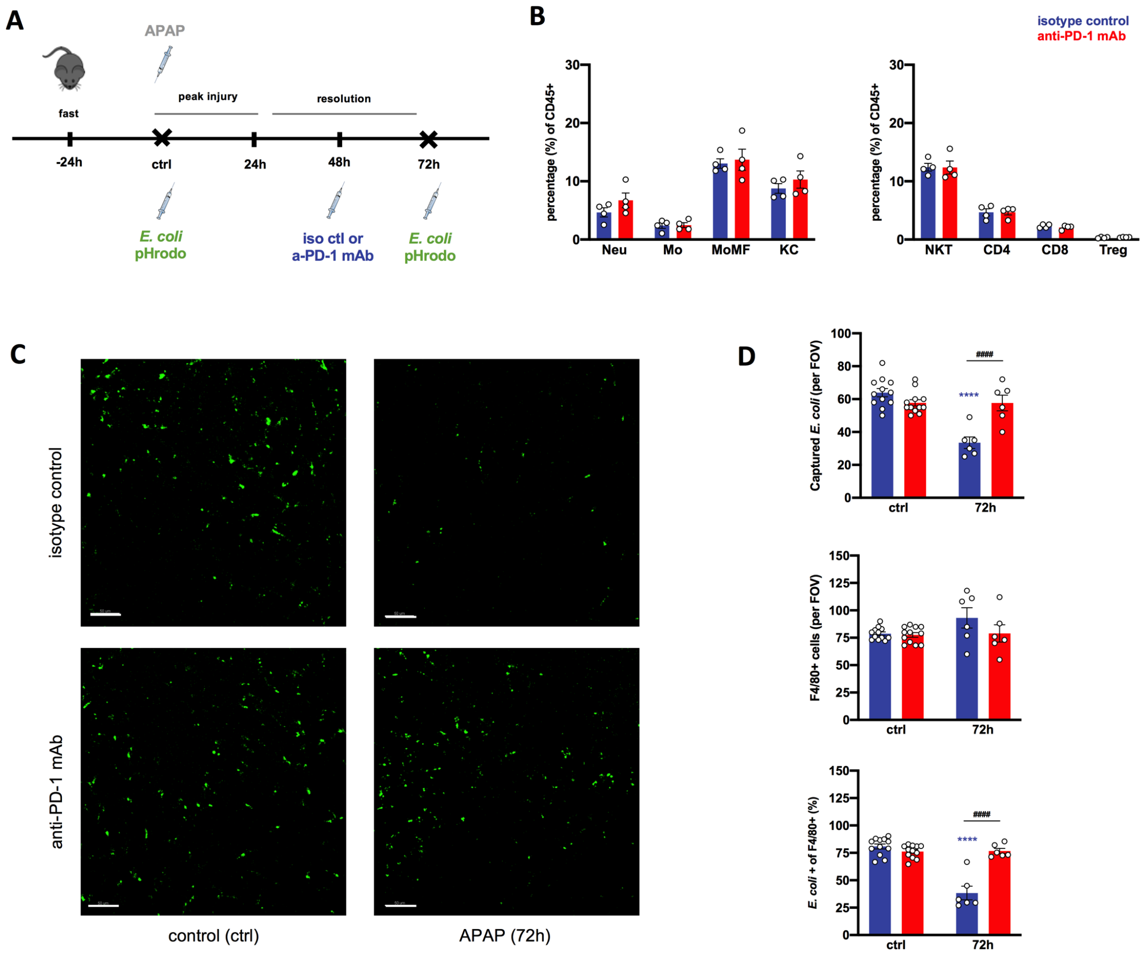

Fig. S7. Anti-PD-1 treatment restores Kupffer cell *E. coli* pHrodo uptake after acute liver injury. Baseline (n=3) or APAP-treated (72h) WT mice (n=2), dosed with isotype control or anti-PD-1 monoclonal antibody (mAb) at 48h post APAP, were challenged with *E. coli* pHrodo. **(A)** Experimental strategy of *E. coli* pHrodo challenge in WT mice. **(B)** Phenotypic characterization of liver CD45+ leukocytes was performed by flow cytometry in WT mice treated with isotype control or anti-PD-1 mAb (n=4 per group). Data show the proportion of neutrophils (Neu), monocytes (Mo), Kupffer cells (KC), monocyte-derived macrophages (MoMF), NKT, CD4+, CD8+ and Treg cells**.** **(C)** Intravital liver images from control and APAP-treated (72h) mice at 20 min post-infection. Scale bars: 50 µm. **(D)** Intravital imaging analysis showing the amount of captured *E. coli* pHrodo, F4/80+ cells and percentage of F4/80+ *E. coli*+ cells per field-of-view (FOV); each dot showing counts per single FOV. Two-way ANOVA test used. Results are from two independent experiments. **** or ####: P< 0.0001.

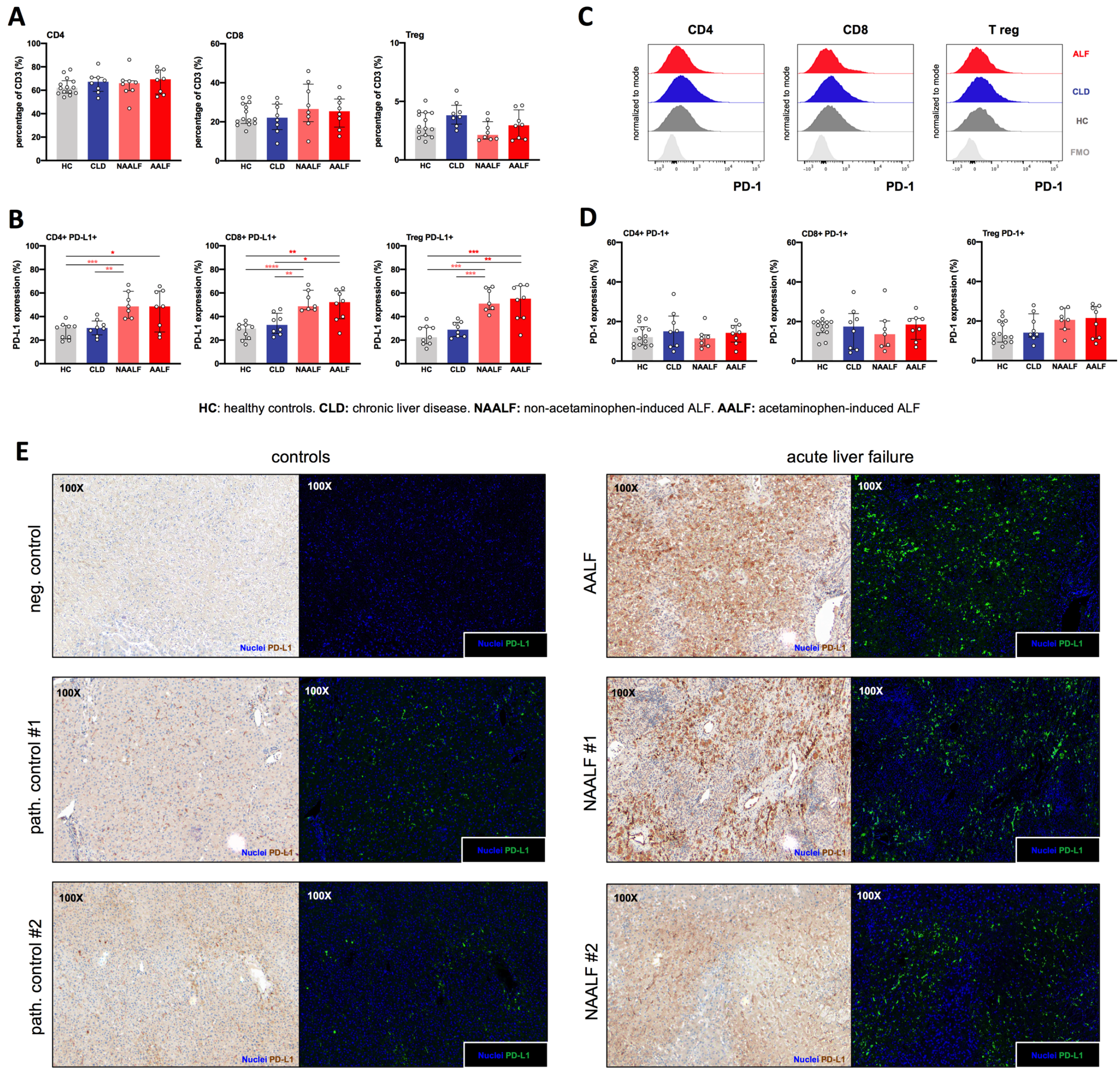

Fig. S8. Peripheral lymphocyte and hepatic PD-L1 expression are increased in human acute liver failure. Phenotypic characterization of lymphocytes was performed using flow cytometry in PBMCs from healthy controls (HC, n=9-14) and patients with chronic liver disease (CLD, n=8) and acute liver failure (ALF, n=16; NAALF: non-acetaminophen-induced ALF, AALF: acetaminophen-induced ALF). **(A)** Data show CD4+, CD8+ and Treg cells as percentage (%) of CD3+ lymphocytes. (**B)** Data show PD-L1 expression (%) of CD4+, CD8+ and Treg cells in different groups. **(C-D)** Representative histogram plots and data showing PD-1 expression (%) of CD4+, CD8+ and Treg cells in different groups. (**E)** Representative immunohistochemistry images of PD-L1 staining in negative control (no primary antibody), pathological control (n=2), AALF (n=1) and NAALF (n=2) liver tissue. Images were analyzed by Nuance™ (PerkinElmer) multispectral technology. Left panels: RGB images show nuclei (blue) and PD-L1 (brown) staining (100X magnification). Right panels: Pseudofluorescent images show nuclei (blue) and PD-L1 (green) staining (100X magnification). NAALF: non-acetaminophen-induced acute liver failure. AALF: acetaminophen-induced acute liver failure. Mann-Whitney test used. Data presented as median values. * P< 0.05, ** P< 0.01, *** P< 0.001, **** P< 0.0001. FMO: fluorescence minus one. MFI: mean fluorescence intensity. SSC: side scatter.

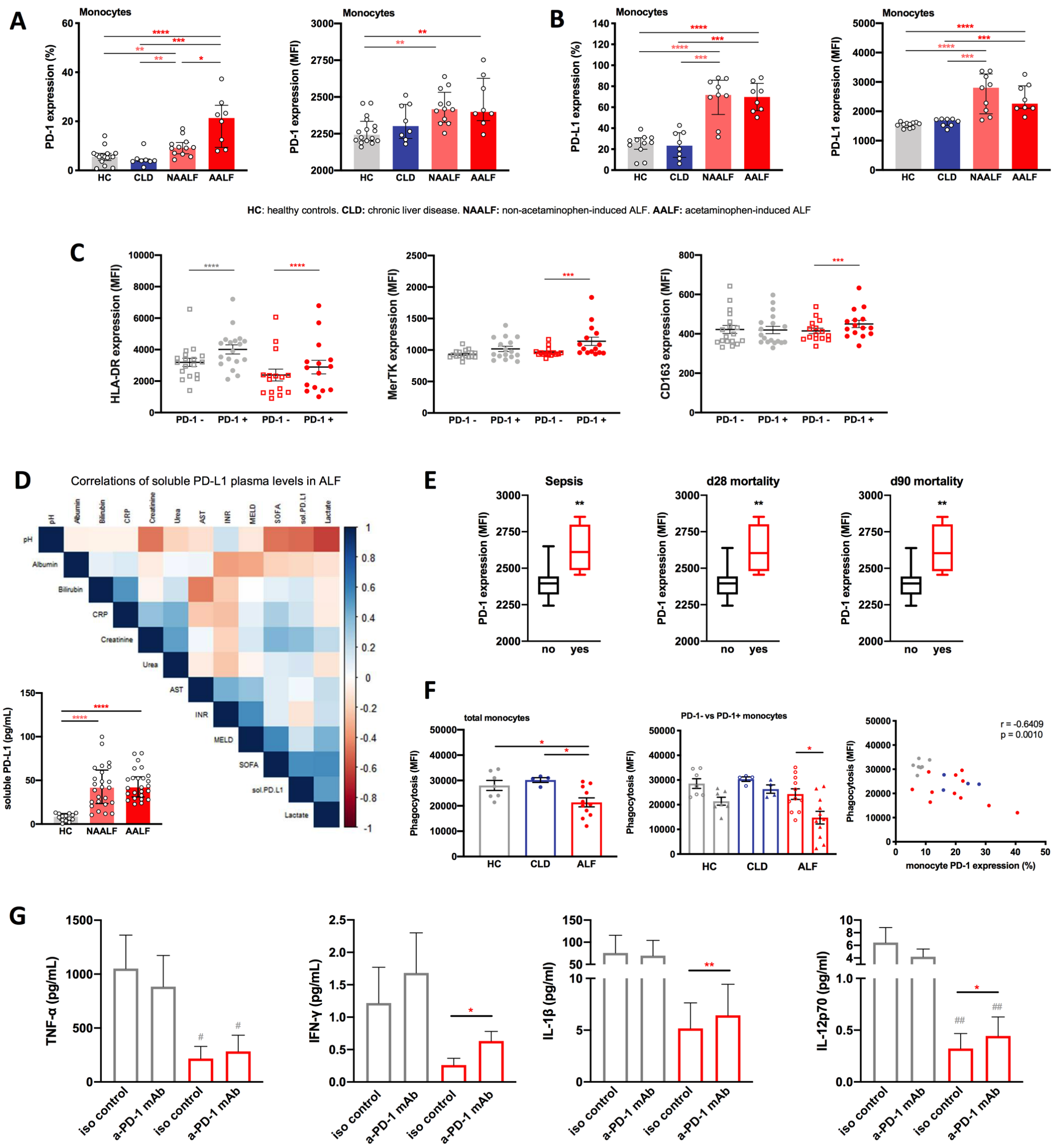

Fig. S9. Phenotypic and functional characterization of peripheral monocytes in human acute liver failure. Phenotypic characterization of monocytes was performed by flow cytometry in PBMCs from healthy controls (HC, n=16), patients with chronic liver disease (CLD, n=8) and acute liver failure (ALF, n=20; NAALF: non-acetaminophen-induced ALF, AALF: acetaminophen-induced ALF). **(A)** PD-1 expression (% and MFI) in total monocytes. **(B)** PD-L1 expression (% and MFI) in total monocytes. **(C)** Phenotypic characterization of PD-1+ vs PD-1- monocyte subsets in HC (grey) and ALF (red); data show HLA-DR, MerTK and CD163 expression levels (MFI). **(D)** Data show the correlations of soluble PD-L1 plasma levels with clinical parameters in patients with ALF (n=50) and soluble PD-L1 plasma levels in different groups (HC: n=14; NAALF: n=25; AALF: n=25). **(E)** Monocyte PD-1 expression (MFI) in ALF patients based on development of sepsis (no: n=16; yes: n=4), day28 mortality (no: n=16; yes: n=4) or day90 mortality (no: n=16; yes: n=4). **(F)** (Left) Monocyte *E. coli* pHrodo phagocytosis was assessed in HC (n=7), CLD (n=4) and ALF (n=11) PBMCs cultured with 20% autologous plasma. (Middle) Quantification of *E. coli* phagocytosis within PD-1+ and PD-1- monocytes in HC, CLD and ALF. (Right) Correlation of monocyte *E. coli* phagocytosis index (MFI) with monocyte PD-1 expression (%) in HC (grey), CLD (blue) and ALF (red). **(G)** HC (n=6) and ALF (n=9) PBMCs were pre-cultured (20% autologous plasma) in the presence of anti-PD-1 monoclonal antibody (a-PD-1 mAb, 10µg/mL) or control (iso control, 10µg/mL) prior to LPS stimulation (100ng/mL). MSD was used to quantify secreted cytokines in cell culture supernatants; Mann-Whitney test used for comparison between different groups; Wilcoxon paired t test was used for comparison within same group. NAALF: non-acetaminophen-induced acute liver failure. AALF: acetaminophen-induced acute liver failure. * P< 0.05, ** P< 0.01, *** P< 0.001, **** P< 0.0001. MFI: mean fluorescence intensity.

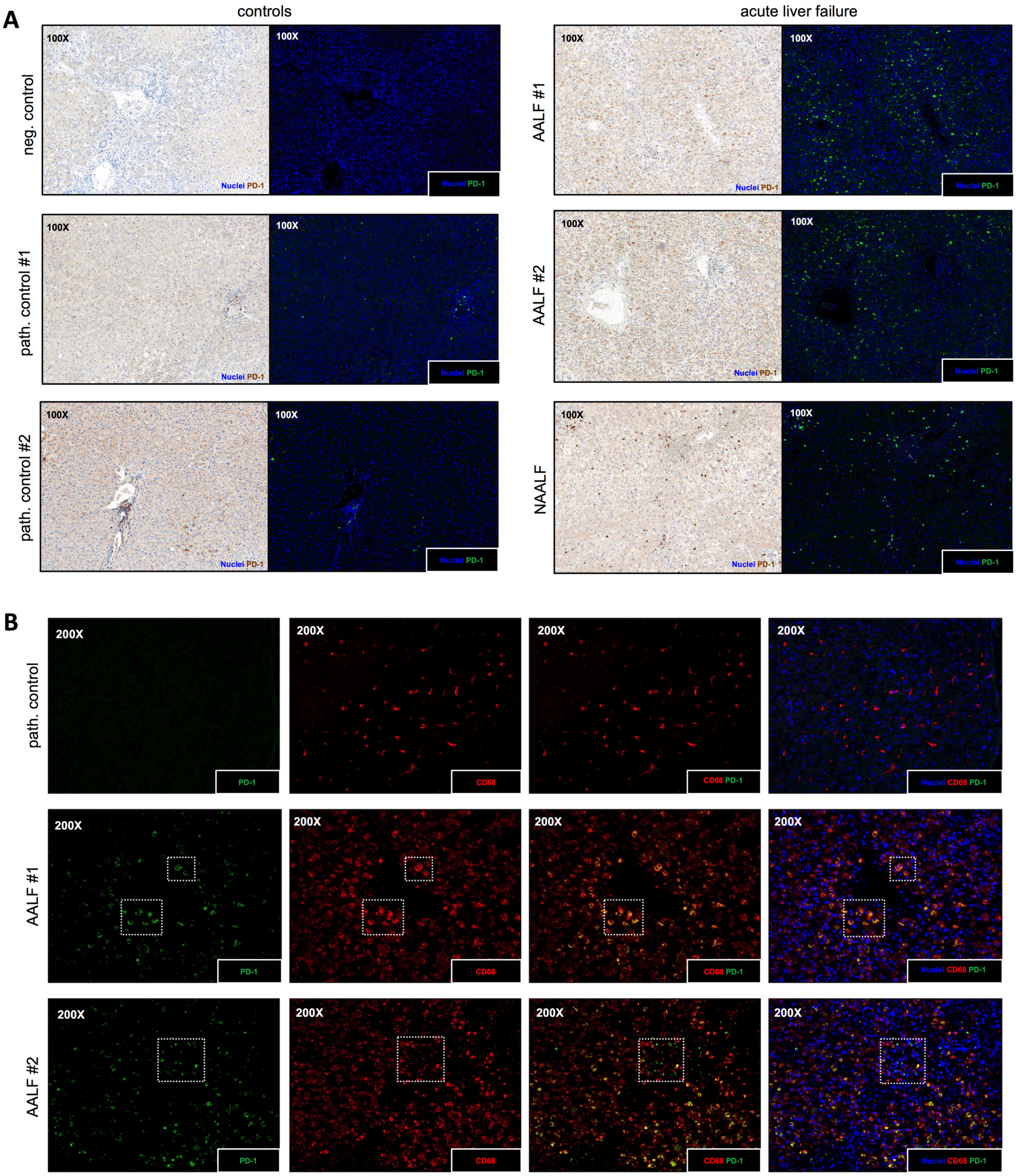

Fig. S10: Hepatic PD-1 expression is increased in human acute liver failure. (A) Representative images of PD-1 immunohistochemistry staining in negative control (no primary antibody), pathological control (n=2), acetaminophen-induced ALF (n=2) and non-acetaminophen-induced ALF (NAALF, n=1) liver tissue. Images were analyzed by Nuance™ (PerkinElmer) multispectral technology. Left panels: RGB images show nuclei (blue) and PD-1 (brown) staining (100X magnification). Right panels: Pseudofluorescent images show nuclei (blue) and PD-1 (green) staining (100X magnification). **(B)** Representative images of PD-1/CD68 double-epitope immunefluorescence staining in pathological control (n=1) and acetaminophen-induced ALF (n=2) liver tissue. Images show PD-1 (green), CD68 (red) and nuclei (blue) staining; PD-1/CD68 co-localization is shown in (yellow) (200X magnification). Image insets (dot-lined squares) indicate PD-1 expression in (second row) $CD68^{+}$ macrophages and (third row) $CD68^{neg}$ cells with lymphocytic morphology. NAALF: non-acetaminophen-induced acute liver failure. AALF: acetaminophen-induced acute liver failure.

Video S1. **Intravital imaging of Kupffer cell bacterial capture in the liver.** Liver intravital imaging was performed in WT mice intravenously challenged with GFP-expressing *E. coli*. Time-lapse video of a mouse at steady-state conditions showing captured *E. coli* (Green, GFP, pseudocolor) by Kupffer cells (Purple, anti-F4/80 labelled, pseudocolor) which reside within liver sinusoids (Blue, anti-CD31 labelled, pseudocolor). Bacteria that remained associated with Kupffer cells for more than 2 min were considered as captured. Time-lapse videos were recorded for up to 20 min post *E. coli* challenge. Scale bars: 50 µm.

Video S2. **3D reconstitution of liver intravital imaging with increasing transparency showing bacteria inside Kupffer cells.** Liver intravital imaging was performed in WT mice intravenously challenged with GFP-expressing *E. coli*. Time-lapse video of a mouse at steady-state conditions shows captured *E. coli* (Green, GFP, pseudocolor) by Kupffer cells (Purple, anti-F4/80 labelled, pseudocolor). 3D reconstitution with increasing transparency of Kupffer cells indicated that the trapped *E. coli* were inside Kupffer cells. Scale bars: 50 µm.

Supplemental Table 1. Clinical and physiological parameters of patients with acute liver failure (ALF) - plasma soluble PD-L1 levels.

Parameters	HC	ALF
**Number (n)**	14	50
**Sex (M/F)**	8 / 6	29 / 21
**Aetiology**	n/a	*Drug-induced: 36*<br>[Acetaminophen: 25<br>Mixed overdose: 11]<br>*Non-drug induced: 14*
**WBC (x10^9/L)**	n/a	8.94 [2.69-23.06]
**Monocytes (x10^9/L)**	n/a	0.22 [0.08-1.96]
**Neutrophils (x10^9/L)**	n/a	7.68 [2.09-21.87]
**Lymphocytes (x10^9/L)**	n/a	0.74 [0.17-3]
**Albumin (g/L)**	n/a	26 [13-36]
**AST (IU/mL)**	n/a	3821 [39-15560]
**Bilirubin (µmol/L)**	n/a	88 [26-786]
**Creatinine (µmol/L)**	n/a	105 [26-462]
**INR**	n/a	3.88 [1-15]
**MELD**	n/a	38.4 [21-45]
**SOFA**	n/a	12 [1-17]

Abbreviations: HC, healthy control; ALF, acute liver failure; INR, international-normalised ratio; AST, aspartate aminotransferase; MELD, Model-for-End-stage-Liver-Disease; SOFA, sequential organ failure assessment; WBC, white blood cell count. Data are presented as median values [range].

B. Supplemental Methods

Mouse Supplemental Methods

Animals

All experimental protocols were approved by Imperial College London Animal Welfare and Ethics Board in accordance with U.K. Home Office regulations. C57BL/6 wild-type (WT) mice were purchased from Charles River Laboratories and PD-1$^{-/-}$ (B6.Cg-*Pdcd1*$^{tm1.1Shr}$/J) mice were purchased from The Jackson laboratory. Mice were housed and bred at Imperial College London animal facility under specific pathogen-free conditions. For induction of sterile acute liver injury, male mice (aged 8-12 weeks) were fasted overnight (12 hours) and received an intraperitoneal injection of 250 mg/kg acetaminophen (APAP) (Sigma-Aldrich) diluted in sterile saline. WT mice received an intraperitoneal injection of either 200 µg anti–PD-1 monoclonal antibody (mAb) (clone RMP1-14, BioXCell) or 200 µg IgG2a control (clone 2A3, BioXCell) diluted in 200µL sterile saline.

Blood and liver tissue sampling

Groups of mice were culled at three time points (24, 48 or 72 hours) after APAP dosing and were compared to baseline (control) animals. Mice were placed under terminal anaesthesia by receiving an intraperitoneal (i.p.) injection of anesthetic overdose. Following confirmation of deep anesthesia, blood was collected via cardiac puncture (right ventricle). Liver function was assessed by measuring alanine transaminase (ALT) levels (Harwell MRC centre, UK) in plasma. The left ventricle was catheterized, an incision was made in the right ventricle and PBS was perfused through the circulation. Visual inspection confirmed blanching of the liver and adequate perfusion. The liver was next excised; the median lobe and gallbladder were separated from the rest of the liver and divided. Half the median lobe was place in formalin for

fixation and half was snap frozen in liquid nitrogen. The remaining liver was transferred into an ice-kept Falcon tube containing PBS and was used for flow cytometry experiments.

H&E staining

Formalin-fixed paraffin embedded (FFPE) liver tissue sections (4 μm thick) were stained with haematoxylin and eosin (H&E) using the following protocol: dewaxing (10 min), rehydration (3 consecutive Et-OH baths with decreasing concentrations followed by a 10 min immersion in distilled water), Harris haematoxylin (5 min), washing (3 min in tap water), acid alcohol (rinse), washing in tap water (3 min), eosin (5 min), washing in tap water (3 min), dehydration (3 different 100% ethanol baths – rinsing), clarification (10 min in xylene), cover-slip with DPX mount medium (Leica). For assessment of liver parenchymal injury, H&E stained FFPE liver tissue sections were imaged, and scoring was carried out by a liver histopathologist. Necrosis score: (0=none), (1=perivenular swelling), (2=perivenular necrosis), (3=bridging necrosis), (4=extensive bridging necrosis), (5=peri-lobular necrosis).

Quantitative reverse transcription PCR (RT-qPCR)

Liver tissue was collected and snap frozen from baseline (control) and APAP-treated mice. Tissues were disrupted in TissueLyser II (Qiagen) and total RNA was extracted by RNeasy Plus Mini Kit (Qiagen). RNA quality and quantity were assessed by OD reading at 260nm and 280nm. One microgram (1 μg) of total RNA was converted to cDNA using SuperScript IV reverse transcriptase with Random Hexamers (Invitrogen). Targeted and housekeeping genes were identified using Ensembl database (Mouse (GRCm38.p5) and RefSeq accession number used to design qPCR primers using NCBI/Primer-BLAST **(Suppl. Table 1).** The qPCR reaction was performed with 100ng of cDNA per reaction in triplicates for each sample using

2xSensiMix SYBR Lo-ROX (Bioline) according to the manufacturer's protocol in AB Viia7-fast block instrument. Relative fold-change in gene expression was calculated as $2^{-\Delta\Delta Ct}$.

mRNA	Accession #	Forward	Reverse
Pdl1	NM_021893.3	AAGTCAATGCCCCATACCGC	CTCTTCCCACTCACGGGTTG
Cxcl9	NM_008599.4	TCGGACTTCACTCCAACACAG	AGGGTTCCTCGAACTCCACA
Cxcl10	NM_021274	TCTGAGTGGGACTCAAGGGAT	AGGCTCGCAGGGATGATTTC
Cxcl12	NM_021704.3	GCTCTGCATCAGTGACGGTA	TAATTTCGGGTCAATGCACA
Cxcl16	NM_023158.7	TCCTTTTCTTGTTGGCGCTG	GGACTGCAACTGGAACCTGATAA
Il10	NM_010548.2	GGTTGCCAAGCCTTATCGGA	GAGAAATCGATGACAGCGCC
Tnfa	NM_013693.3	GATCGGTCCCCAAAGGGATG	GGTGGTTTGCTACGACGTG
Arg-1	NM_007482	GTACATTGGCTTGCGAGACG	TTTTCTTCCTTCCCAGCAGGT
Nos2	NM_010927	GAAACTTCTCAGCCACCTTGG	TCCAACGTTCTCCGTTCTCTTG
18S	n/a	GCAATTATTCCCCATGAACG	GGCCTCACTAAACCATCCAA
Gapdh	NM_001289726.1	CATCACTGCCACCCAGAAGACTG	ATGCCAGTGAGCTTCCCGTTCAG

Supplemental Table 2. Primers used for quantitative reverse transcription PCR (RT-qPCR) in WT and PD-1 KO mice.

Kupffer cell bacterial uptake assay

For *in vivo* imaging of pHrodo™ Green *E. coli* BioParticles® uptake by macrophages, WT mice received intravenous injection of sterile PBS containing 250 µg of pHrodo™ Green *E. coli* BioParticles® (2mg/mL, #P35366, Life Technologies) and anti-F4/80-BV421 (5µg/mouse; clone BM8, Biolegend) antibody prior to mouse liver preparation. Mice were imaged using an inverted Leica TCS SP5 Confocal microscope with DM6000 Confocal Fixed Stage (Leica).

Inflammatory markers and cytokines

Plasma lactate was measured using an L-Lactate Assay kit (# ab65331, Abcam) and C-reactive protein (CRP) was measured using an enzyme-linked immunosorbent assay (# MCRP00, R&D Systems), according to manufacturer's instructions. Plasma cytokines (TNF-α, IL-6 and IL-10) were measured by bead-based multiplex assay using a LEGENDplex™ Mouse Inflammation Panel (# 740446, BioLegend).

Isolation of liver leukocytes

Isolation of mouse liver non-parenchymal cells was performed. Livers were harvested from PBS-perfused mice, chopped finely and incubated for 45 min with digestion buffer [5% fetal bovine serum (Gibco), 0.5 mg/mL Collagenase VIII from *Clostridium histolyticum* (Sigma-Aldrich, UK), 0.1 mg/mL Deoxyribonuclease I from bovine pancreas (Sigma-Aldrich, UK) in Dulbecco's PBS with calcium and magnesium (Gibco)] in a 15 mL. Falcon tubes on a shaker-incubator at 250 rpm (37ºC, 5% CO2). After incubation, single cell suspensions were filtered over a 100 µm filter (BD Biosciences) attached to 50 mL. Falcon tubes. The samples were next subjected to two cycles of washing with Dulbecco's PBS without calcium and magnesium (Gibco) at 400 rpm, 4°C for 5 min from which the supernatant was kept, omitting the parenchymal cell pellet. Subsequently, the supernatant was centrifuged at 1.400 rpm, 4°C for

5 min and the cell pellet was lysed for blood erythrocytes by 2 min room temperature incubation with ACK lysing buffer (Gibco) followed by a was with PBS.

Flow cytometry of blood immune cells

Circulating blood samples were taken by cardiac puncture and collected in blood collection tubes (Microvette, Sarstedt) to prevent clotting. Blood samples (100 µL) were added into FACS tubes and lysed in 2 mL RBC Lysis Buffer (1X) (Invitrogen) for 10 min at room temperature in the dark. After incubation, FACS tubes were spun down (5 min, 500g at room temperature) and cells were resuspended in 100 µL FACS buffer (PBS with 2% FBS). All samples were pre-incubated for 10 min at room temperature with TruStain fcX™ (anti-mouse CD16/32) antibody (BioLegend) and then stained for 25 min at room temperature in the dark with monoclonal antibodies detailed in (**Suppl. Table 2**). Samples were washed with PBS, spun down (5 min, 500g at room temperature) and resuspended in 150 µL FACS buffer and 50 µL 123count™ eBeads Counting Beads (Invitrogen). Acquisition was performed on BD LSRFortessa™ cell analyzer (BD Biosciences). Flow cytometry data analysis was performed in FlowJo™ v10 (Becton Dickinson & Company). Data graph presentation and statistical analyses were performed in Prism 8 (GraphPad Software).

Flow cytometry of liver immune cells

Liver leukocytes were isolated as described above. Following ACK lysing buffer (Gibco) incubation and PBS wash, cells were transferred into FACS tubes, resuspended in 100 µL FACS buffer (PBS with 2% FBS). All samples were pre-incubated 10 min at room temperature with TruStain fcX™ (anti-mouse CD16/32) antibody (BioLegend) prior to staining for 25 min (room temperature, in the dark) with monoclonal antibodies detailed in (**Suppl. Table 3 and 4**). Samples were washed with PBS (5 min, 500g) and resuspended in 150 µL FACS buffer

(Invitrogen) and 50µL 123count™ Counting Beads (Invitrogen). Acquisition was performed on BD LSRFortessa™ cell analyzer (BD Biosciences). Flow cytometry data analysis was performed in FlowJo™ v10 (Becton Dickinson & Company). Data graph presentation and statistical analyses were performed in Prism 8 (GraphPad Software).

Marker	Fluorochrome	Company	Catalog #
Ly6G	BV605	BioLegend	127639
CD45	BV650	BioLegend	103151
CD11b	BV711	BioLegend	101242
CD3	BV785	BioLegend	100232
CD8	FITC	Invitrogen	11-0081-85
NK1.1	PE	BioLegend	108708
Ly6C	PE-Cy7	BioLegend	128018
CD4	APC	BioLegend	100424
TCR-β	APC-Cy7	BioLegend	109220

Marker	Fluorochrome	Company	Catalog #
CD11b	BV711	BioLegend	101242
CD45	BV650	BioLegend	103151
CD64	PerCP-Cy5.5	BioLegend	139308
F4/80	BV421	BioLegend	123137
Ly6C	PE-Cy7	BioLegend	128018
Ly6G	BV605	BioLegend	127639
MHCII	APC eFluor780	Invitrogen	47-5321-82
PD-1	PE	BioLegend	135206
PD-L1	BV785	BioLegend	124331

Supplemental Table 3 and 4. Anti-mouse monoclonal antibodies used for the phenotypic characterization of mouse blood immune and liver myeloid cells. APC, allophycocyanin; BV, Brilliant Violet; FITC, fluorescein isothiocyanate; PE, phycoerythrin.

Marker	Fluorochrome	Company	Catalog #
CD25	PE	BioLegend	102008
CD3	BV785	BioLegend	100232
CD4	AF647	BioLegend	100424
CD45	BV650	BioLegend	103151
CD8	BV711	BioLegend	100759
CXCR6	FITC	BioLegend	151108
FoxP3	AF488	BioLegend	126406
NK1.1	PE-Cy7	BioLegend	108714
PD-1	BV421	BioLegend	135221
PD-L1	BV605	BioLegend	124321
TCR-β	PerCP-Cy5.5	BioLegend	109228

Supplemental Table 5. Anti-mouse monoclonal antibodies used for the phenotypic characterization of mouse liver lymphoid cells. APC, allophycocyanin; AF, Alexa Fluor; BV, Brilliant Violet; FITC, fluorescein isothiocyanate; PE, phycoerythrin.

Kupffer cell (Clec4F) tissue staining

Mouse FFPE liver tissue (4 µm) sections were deparaffinized, rehydrated and subjected to heat-induced epitope retrieval (HIER) using Tris EDTA pH 9 solution (20 min). Sections were incubated with CAS-Block™ protein block (# 00-8120; Invitrogen) and with dual-endogenous enzyme block (# S2003; Agilent Technologies) at room temperature, followed by overnight incubation (fridge) with goat anti-mouse CLEC4F/CLECSF13 antibody (1:50 dilution; # AF2784; R&D systems). Slides were then washed with PBS and incubated with Goat IgG Horseradish Peroxidase (HRP) conjugated polymer (1:500 dilution; # HAF017; R&D systems). The signal was detected using the EnVision™ G|2 doublestain system, rabbit/mouse (DAB+/permanent red) kit (Agilent Technologies). After counterstaining with hematoxylin, sections were dehydrated, cleared with xylene, cover slipped and mounted with DPX (Leica).

WT mice	Liver (g)	PD-1 KO mice	Liver (g)
**WT - ctrl -1**	0.8	**PD-1 KO - ctrl -1**	1.0
**WT - ctrl -2**	0.9	**PD-1 KO - ctrl -2**	1.0
**WT - ctrl -3**	0.9	**PD-1 KO - ctrl -3**	0.8
**WT - ctrl -4**	0.9	**PD-1 KO - ctrl -4**	0.9
**WT - A24h -1**	0.9	**PD-1 KO - A24h -1**	1.0
**WT - A24h -2**	0.8	**PD-1 KO - A24h -2**	0.7
**WT - A24h -3**	0.8	**PD-1 KO - A24h -3**	0.7
**WT - A24h -4**	0.7		
**WT - A48h -1**	0.6	**PD-1 KO - A48h -1**	1.0
**WT - A48h -2**	0.9	**PD-1 KO - A48h -2**	0.8
**WT - A48h -3**	0.9	**PD-1 KO - A48h -3**	0.8
**WT - A48h -4**	0.7		
**WT - A72h -1**	0.8	**PD-1 KO - A72h -1**	0.9
**WT - A72h -2**	0.9	**PD-1 KO - A72h -2**	0.8
**WT - A72h -3**	0.8	**PD-1 KO - A72h -3**	0.7
**WT - A72h -4**	0.7	**PD-1 KO - A72h -4**	0.7

Supplemental Table 6. Liver tissue weights of wild-type (WT) and PD-1-deficient (PD-1 KO) mice used for characterization of myeloid and lymphoid cell subsets by flow cytometry before (control) and after APAP-induced acute liver injury. Liver tissue sampling for flow cytometry experiments was performed as described above (page 18).

WT mice	Liver (g)	PD-1 KO mice	Liver (g)
**WT - ctrl -1**	1.2	**PD-1 KO - ctrl -1**	1.0
**WT - ctrl -2**	1.4	**PD-1 KO - ctrl -2**	1.4
**WT - ctrl -3**	1.1	**PD-1 KO - ctrl -3**	1.2
**WT - ctrl -4**	1.2	**PD-1 KO - ctrl -4**	1.2
**WT - ctrl -5**	1.3		
**WT - A72h -1**	1.2	**PD-1 KO - A72h -1**	1.3
**WT - A72h -2**	1.2	**PD-1 KO - A72h -2**	1.4
**WT - A72h -3**	1.4	**PD-1 KO - A72h -3**	1.4
**WT - A72h -4**	1.0	**PD-1 KO - A72h -4**	1.1
		**PD-1 KO - A72h -5**	1.0
		**PD-1 KO - A72h -6**	1.3
**WT - A72h - iso ctrl -1**	1.1	**WT - A72h - anti-PD-1 -1**	1.1
**WT - A72h - iso ctrl -2**	1.2	**WT - A72h - anti-PD-1 -2**	1.3
**WT - A72h - iso ctrl -3**	1.1	**WT - A72h - anti-PD-1 -3**	1.4
		**WT - A72h - anti-PD-1 -4**	1.2

Supplemental Table 7. Liver tissue weights of wild-type (WT) and PD-1-deficient (PD-1 KO) mice used in the systemic *Escherichia coli* challenge (20 min post-infection) experiments.

Human Supplemental Methods

Flow cytometry of human immune cells

Whole blood was taken and used to isolate human peripheral blood mononuclear cells (PBMCs) by density gradient centrifugation layered on Ficoll-Paque Plus (GE Healthcare). Human PBMC were thawed, washed with PBS, counted and incubated with live/dead staining and a mix of monoclonal antibodies detailed in (**Suppl. Table 7 and 8**) for 20 min (room temperature, in the dark). Samples were washed with PBS (5 min, 1.800 rpm) and resuspended in 150 µL FACS buffer (Invitrogen) and 50µL 123count™ Counting Beads (Invitrogen). Acquisition was performed on BD LSRFortessa™ cell analyzer (BD Biosciences). Flow cytometry data analysis was performed in FlowJo™ v10 (Becton Dickinson & Company). Data graph presentation and statistical analyses were performed in Prism 8 (GraphPad Software).

Soluble PD-L1 ELISA

Human PD-L1 enzyme-linked immunosorbent assay (# BMS2212, Invitrogen) was used to determine plasma soluble PD-L1 levels in healthy controls and patients with acute liver failure. Samples were run in duplicates and their 450nm absorbance (OD) was read on a Sector Imager 2400 apparatus (Gaithersburg). Experiments were performed according to the manufacturer's instructions.

Mesoscale Discovery (MSD)

We used a Mesoscale Discovery Human Pro-inflammatory 10-plex panel (MSD) to determine cytokine levels in cell culture supernatants. Plates were read on Sector Imager 2400 apparatus (Gaithersburg). Experiments were performed in duplicates and according to the manufacturer's instructions.

Monocyte treatment with anti-PD-1 blocking antibody

For assessment of monocyte phagocytosis, PBMCs isolated from healthy controls and acute liver failure patients were cultured in 48-well flat-bottom plates in medium [RPMI-1640 medium with 10% FBS and 1% antibiotics (L-glutamine, penicillin, and streptomycin) (Gibco)] containing 20% autologous plasma. PBMCs were cultured in the presence of 10μg/mL anti-PD-1 monoclonal antibody (mouse anti-human CD279 (PD-1) monoclonal antibody, clone J116, Invitrogen) or isotype control (mouse IgG1 kappa isotype control, clone P3.6.2.8.1, Invitrogen) prior to phagocytosis (37°C, 5% CO_2). PBMCs were incubated with 10 μl of pHrodo™ Green *E. coli* BioParticles® (2mg/mL, # P35366, Life Technologies) for 60min 37°C, 5% CO_2. Following incubation, phagocytosis was stopped by leaving tubes on ice for 10 min. Cells were then washed with PBS and stained with anti-human monoclonal antibodies (CD14 and HLA-DR) in FACS buffer for 20 min (fridge, dark). Cells were washed and re-suspended in FACS buffer prior to acquisition on a BD LSRFortessa™ cell analyzer (BD).

Tissue immunohistochemistry

Formalin-fixed paraffin-embedded (FFPE) liver tissue (4 μm) sections were deparaffinized, rehydrated and subjected to heat-induced epitope retrieval (HIER) using EDTA pH 9 solution (20 min). Endogenous peroxidase activity was quenched with 3% hydrogen peroxide. Sections were incubated with rabbit monoclonal anti-PD-L1 antibody (1:100 dilution; # 13684, Cell Signaling) or rabbit monoclonal anti-PD-1 antibody (1:500 dilution; # ab137132, Abcam) and subsequently incubated with anti-rabbit IgG horseradish peroxidase (HRP) conjugated polymer (# DS9800, Leica Biosystems). After signal detection with DAB as chromogen, sections were dehydrated, counterstained with hematoxylin and mounted with DPX (Leica Biosystems). Images were captured with a Nikon Eclipse E600 microscope and processed with Nuance™ (PerkinElmer) multispectral imaging technology.

Tissue fluorescent staining

Human FFPE liver tissue (4 μm) sections were deparaffinized, rehydrated and subjected to HIER using Tris EDTA buffer pH9 solution (20 min). Sections were incubated for one hour (room temperature) with 5% goat serum PBS (blocking non-specific binding) and subsequently incubated overnight (4°C, fridge) with a rabbit monoclonal anti-PD-1 antibody (1:250 dilution; # ab137132, Abcam). Sections were next washed with PBS (3X), incubated for 45 min (room temperature) with a goat anti-rabbit IgG (H+L) AF488-conjugated secondary antibody (1:500 dilution; # A11034, Invitrogen) and washed with PBS (3X) again. Sections were next incubated for 45 min (room temperature) with a mouse monoclonal anti-CD68 antibody (ready-to-use; # IR61361-2, Agilent Technologies), washed with PBS (3X) and subsequently incubated for 45 min (room temperature) with a goat anti-mouse IgG (H+L) AF555-conjugated secondary antibody (1:500 dilution; # A32727, Invitrogen). Finally, sections were washed with PBS (3X), mounted with fluoroshield with DAPI (Sigma-Aldrich) and stored in the dark (4°C). Images were captured using the Leica DM4 B microscope and processed with the Leica Application Suite (LAS) X 3.3 software (Leica).

Marker	Fluorochrome	Company	Catalog #
CD14	PE-Cy7	BD Biosciences	557742
CD16	APC-H7	BD Biosciences	560195
CD163	PE	BD Biosciences	556018
FVD	eFluor 506	Invitrogen	65-0866-14
HLA-DR	PerCP-Cy5.5	Invitrogen	45-9956-42
MerTK	APC	R&D Systems	FAB8912A
PD-1	BV786	BD Biosciences	563789
PD-L1	BV605	BD Biosciences	329724

Supplemental Table 8. Anti-human monoclonal antibodies used for the phenotypic characterization of human blood lymphocytes. APC, allophycocyanin; BV, Brilliant Violet; FITC, fluorescein isothiocyanate; FVD, Fixable Viability Dye: PE, phycoerythrin.

Marker	Fluorochrome	Company	Catalog #
CD4	APC-Cy7	BioLegend	344616
CD127	PE-Cy7	Invitrogen	25-1278-42
CD25	PE-CF594	BD Biosciences	562403
CD3	eFluor 450	Invitrogen	48-0036-42
CD8	APC	Invitrogen	17-0088-42
FVD	eFluor 506	Invitrogen	65-0866-14
PD-1	BV786	BD Biosciences	563789
PD-L1	BV605	BD Biosciences	329724

Supplemental Table 9. Anti-human monoclonal antibodies used for the phenotypic characterization of human blood lymphocytes. APC, allophycocyanin; BV, Brilliant Violet; FITC, fluorescein isothiocyanate; FVD, Fixable Viability Dye: PE, phycoerythrin.
